# Size-dependent dispersion characteristics of piezoelectric sandwich nanoplates taking surface effects into account

**DOI:** 10.1038/s41598-025-01789-3

**Published:** 2025-05-17

**Authors:** Biao Hu, Juan Liu, Aizhong Luo, Qi Li, Yuxing Wang, Jing Wang, Huoming Shen

**Affiliations:** 1 School of Civil Engineering, Guizhou University of Engineering Science, Bijie, 551700 China; 2 School of Mechanics and Aerospace Engineering, Southwest Jiaotong University, Chengdu, 610031 China; 3 School of Mechanical and Electrical Engineering, Southwest Petroleum University, Chengdu, 610500 China

**Keywords:** Surface effects, Scale effect, Viscoelastic effect, Piezoelectric sandwich nanoplate, Dispersion property, Mechanical engineering, Electronic properties and materials

## Abstract

In this study, the dispersion behavior of piezoelectric sandwich nanoplates is examined using the nonlocal strain gradient theory (NSGT). Specifically, the evolution law of frequency and phase velocity (PV) with the wave number (WN) is investigated. The sandwich nanoplate has a metallic central layer and a piezoelectric surface layer, and the nanoplate is deposited on top of a viscoelastic substrate. The viscoelastic substrate is modeled using a three-parameter viscoelastic model. The surface effects (SEs) of the piezoelectric layer, including its elastic parameters, piezoelectric parameters, dielectric parameters, and residual stresses, on the dispersion characteristics are systematically considered. The equations of motion are determined by Hamilton’s principle and NSGT. Furthermore, the scale effects, SEs, and viscoelastic effects on the dispersion properties are comprehensively explored. The results reveal that the contribution of scale and viscoelastic effects to the dispersion properties is strongly dependent on the WN, and the impact of SEs on the frequency is inseparable from the thickness of the piezoelectric sandwich nanoplates.

## Introduction

Traditional continuum mechanics assumes materials are defect-free and microscopically continuous. This assumption holds validity only when structural dimensions far exceed defect scales; however, when structural and defect sizes are comparable, the resulting inaccuracies render the theory inadequate. This discrepancy, termed the size-dependent (scale) effect, critically influences the mechanical behavior of micro/nanostructures. Conventional continuum frameworks fail to account for such effects due to the absence of intrinsic length-scale parameters—a limitation that underscores the need for advanced theoretical approaches. To address this issue, nonclassical continuum mechanics was established, which includes the nonlocal elasticity theory (NET), strain gradient theory (SGT), and nonlocal strain gradient theory (NSGT). These theories have been extensively utilized to explore the static-dynamic properties of microstructures and nanostructures. In particular, the analysis of piezoelectric nanostructures based on the scale-dependent theory has garnered considerable research attention.

With the rapid development of nanotechnology, piezoelectric nanostructures are being extensively applied in signal propagation, memory storage, energy storage, and power generation. Several researchers have investigated the mechanical properties of piezoelectric nanostructures using various scale-dependent theories. Based on the NET, the laws of scale effects on the mechanical behavior of piezoelectric nanostructures have been revealed. NET has been applied to systematically analyze the vibrational properties of various piezoelectric nanostructures^1–4^. In addition, the coupled stress theory has been used to examine various mechanical properties of piezoelectric nanostructures. Besides, SGT has been used for analyzing the bending^5^, free vibration^6^, nonlinear vibration^7^, and dynamic instability of piezoelectric nanostructures^8^. The above studies have revealed that the nonlocal parameter (NP) and the length scale parameter (LSP) have an important effect on the bending, buckling, and dynamic properties of piezoelectric nanostructures. Unfortunately, experimental studies have shown that the scale effect of nanostructures can manifest in not only softening but also hardening effects. To address this issue, Lim et al.^9^ proposed the NSGT, which includes nonlocal elastic and strain gradient fields to comprehensively consider the scale effects.

The NSGT has been extensively utilized to examine the mechanical characteristics of piezoelectric nanostructures. For example, considering the nonlocal strain gradient effect and the flexural electric effect, Zeng et al.^10^ examined the nonlinear force-electric nature of piezoelectric nanobeams. Fateme et al.^11^ explored the vibration and dynamic instability of piezoelectric sandwich nanobeams, and Zhou et al.^12^ analyzed the force-electric response of piezoelectric nanoplates. In addition, the vibration characteristics of functionally graded piezoelectric nanoplates have been analyzed based on the NSGT^13^. This theory has also been used to examine the bending properties of functionally graded multilayer piezoelectric nanoplates^14^ and the wave propagation in piezoelectric nanotubes considering viscous fluid transport^15^. Further, Al-Furjan et al.^16^ investigated the kinetic properties of rapidly rotating piezoelectric nanodiscs using the NSGT. Meanwhile, Arefi et al.^17^ explored the dynamic instability region of piezoelectric sandwich nanobeams with surface sheets reinforced by functionally graded carbon nanotubes (CNTs). Studies demonstrate that the bending, vibrational response, nonlinear dynamics, and dynamic instability of piezoelectric nanostructures are governed by the ratio of intrinsic scale parameters. Notably, the scale effects influencing nanostructural wave behavior exhibit multifactorial dependence, including structural dimensions, rotational velocity, gradient indices, and wave number (WN). This underscores the need for a systematic investigation into how scale-dependent mechanisms shape dispersion phenomena in piezoelectric sandwich structures. Once the structural size is reduced to the nanometer scale, it not only exhibits scale effects but also surface effects (SEs). The SE is caused by a significant increase in the surface-to-volume ratio of the nanostructures. With the development of piezoelectric nanomaterials, a series of studies have been conducted on SEs using various scale-dependent theories. Ebrahimi et al.^18^ used the nonlocal theory (NT) to examine the bending features of piezoelectric microbeams under hygrothermal loading considering SEs. Meanwhile, nonlocal surface elasticity theory, bivariate refined plate theory, and NET have been utilized to analyze the buckling characteristics of piezoelectric nanobeams under the action of magnetic field^19^, the uniaxial and biaxial buckling of piezoelectric nanoplates considering surface stresses and NP^20^, and the effect of surface parameters on the flexural behavior of piezoelectric nanoshells^21^, respectively. In addition, the vibrational properties and fluctuation behavior of piezoelectric nanostructures have been analyzed using NT. Considering non-local effects and SEs, Kachapi et al.^22^ discussed the dynamic behavior of piezoelectric nanoresonators. Wang et al.^23^ investigated the dynamic features of piezoelectric nanocircular plates. Ebrahimi et al.^24^ explored the thermal oscillations of piezoelectric nanobeams considering flexoelectric effects. Hosseini-Hashemi et al.^25^ examined the free oscillation of functionally graded piezoelectric nanobeams, and Salman et al.^26^ systematically analyzed the bending, flexural vibrations, and oscillation of piezoelectric nanobeams under the action of magneto-electrical thermodynamic forces. Further, the wave propagation characteristics of piezoelectric nanostructures have been explored. For example, it has been demonstrated that the SEs and nonlocal elasticity can impact the wave propagation properties in piezoelectric-piezomagnetic bilayer nanostructures^27^, functionally graded piezoelectric nanobeams^28^, and piezoelectric nanoplates^29^. Notably, Ebrahimi et al.^30^ investigated wave propagation in piezoelectric nanoplates with surface effects using the nonlocal strain gradient theory. Apart from the NT, the SGT and NSGT have also been utilized to examine the dynamic properties of piezoelectric nanostructures. Khabaz et al.^31^ used the SGT to explore the impact of SEs on the vibrational and dynamic properties of piezoelectric sandwich cantilever nanobeams, and Mohammadimehr et al.^32^ characterized the influence of surface stress on the kinetic stability of piezoelectric polymer nanoplates reinforced by CNTs. Moreover, Amiri et al.^15^ examined the effect of surface stress and Reynolds number on the dispersion properties of viscous-fluid-conveying piezoelectric nanotubes using the NSGT. Studies reveal that scale effects (SEs) critically enhance the stiffness of piezoelectric nanostructures, modulating their bending behavior, flexural vibrations, and dynamic response. These SE-driven alterations are governed by vibration modes and intricately linked to wave numbers (WNs) in dispersion phenomena. Consequently, a systematic investigation into the combined influence of nonlocal strain gradient effects and SEs on wave propagation in piezoelectric sandwich nanoplates is imperative to unravel their scale-dependent electromechanical coupling.

Piezoelectric nanostructures, such as piezoelectric nanobeams, piezoelectric nanoplates, and piezoelectric nanoshells comprising sensors, actuators, and resonators, often need to be embedded in the substrate. Numerous studies have been conducted on the mechanical properties of piezoelectric nanostructures resting on elastic and viscoelastic foundations. For example, considering elastic foundations, Ansari et al.^33^ investigated the scale-dependent bending and buckling of piezoelectric nanobeams incorporating flexoelectric effects. Fatemeh et al.^34^, Yang et al.^35^, and Zenkour et al.^36^ studied the buckling and static response of piezoelectric sandwich microplates under multifield coupling. Further, Wang et al.^37^ and Hong et al.^38^ evaluated the static bending characteristics of functionally graded piezoelectric microplates. In addition, the free vibration characteristics of piezoelectric sandwich microplates^39^, the vibration characteristics of functionally graded piezoelectric microbeams^40^, and the dynamic stability of piezoelectric sandwich microbeams^41^ have been thoroughly explored by using a two-parameter elastic substrate. Furthermore, the nonlinear vibration characteristics of piezoelectric functionally graded microbeams^42^, piezoelectric sandwich nanobeams^43^, and functionally graded piezoelectric nanoshells^44^ under an external electric field have also received considerable research attention. Notably, Sahmani et al.^45–47^ conducted a systematic analysis of the nonlinear mechanical response in piezoelectric intercalated nanocomposites—including sinusoidal pulse-actuated porous nanoplates, CNT-agglomerated beams, and pristine intercalated nanoplates—employing GM-based meshfree collocation formulations. In the above studies, the static-dynamic properties of piezoelectric nanostructures under different elastic substrate parameters were mainly considered. However, the substrates of actual micro- and nanostructures are often viscous, so it is necessary to examine the impact of viscoelastic substrates on the mechanical performance of the structures. For example, Saeed et al.^48^ explored the vibrational characteristics of piezoelectric nanotubes in the presence of a magnetic field, Azhdarzadeh et al.^49^ discussed the nonlinear thermoelastic behavior of fluid-conveying piezoelectric microtubes, Zhang et al.^50,51^ analyzed the thermo-electric power vibration problems of piezoelectric nanobeams and piezoelectric nanoplates, respectively, and Wang et al.^52^ and Li et al.^53^ investigated the wave propagation behavior of functionally graded piezoelectric nanoshells and porous functionally graded piezoelectric nanotubes, respectively. The above studies indicated that the viscoelastic substrate not only affects the bending and flexural properties of the nanostructures, but also has a significant effect on the vibrational frequencies of different modes. The existing studies on the response of functionally graded piezoelectric nanostructures resting on viscoelastic substrates have suggested that the effects of substrate parameters on the frequency is closely related to the WN. However, the dispersion properties of piezoelectric sandwich structures on viscoelastic substrates have been rarely investigated, and this systematic analysis is necessary to boost the application of piezoelectric multilayer films.

Overall, although numerous studies have been conducted on the surface and viscoelastic effects of piezoelectric nanostructures based on various scale-dependent theories, a systematic investigation of the impact of SEs and viscoelastic foundations on the wave propagation characteristics of piezoelectric sandwich nanoplates based on nonlocal strain gradient theories has not been conducted yet, which forms the motivation of this study. The governing equations are derived based on the Hamilton’s principle and sinusoidal shear deformation theory. Further, the equations of motion are deduced using the NSGT, and the characteristic equations are presented based on the harmonic solution. The impact of scale parameters, surface parameters, and viscoelastic parameters on the frequency profile and phase velocity (PV) profile are numerically investigated.

## Theoretical modeling

### Model description

**Fig. 1:**
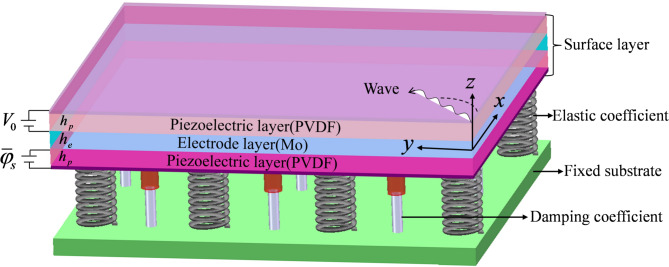
Modeling of sandwich nanoplates.

A model of the sandwich nanoplate is illustrated in Fig. 1. The piezoelectric and metal layers of the nanoplates are assumed to be perfectly bonded. The piezoelectric layer thickness and metal layer thickness are 10 nm and 5 nm, respectively. In addition, the superscripts *e* and *p* in the text represent the metal and piezoelectric layers, respectively.

### Stress-strain relationship

The constitutive equation of a metal is given as follows^54^:

1 $$\left\{ \begin{gathered} \sigma _{{xx}}^{e} \hfill \\ \sigma _{{yy}}^{e} \hfill \\ \sigma _{{xz}}^{e} \hfill \\ \sigma _{{yz}}^{e} \hfill \\ \sigma _{{xy}}^{e} \hfill \\ \end{gathered} \right\}=\left[ {\begin{array}{*{20}{c}} {{p_{11}}}&{{p_{12}}}&0&0&0 \\ {{p_{12}}}&{{p_{22}}}&0&0&0 \\ 0&0&{{p_{55}}}&0&0 \\ 0&0&0&{{p_{44}}}&0 \\ 0&0&0&0&{{p_{66}}} \end{array}} \right]\left\{ \begin{gathered} {\varepsilon _{xx}} \\ {\varepsilon _{yy}} \\ {\gamma _{xz}} \\ {\gamma _{yz}} \\ {\gamma _{xy}} \\ \end{gathered} \right\}$$

where ${p_{ij}}$ represents the elastic constant of the metal. The intrinsic equation of a piezoelectric material is^55^

2 $$\left\{ \begin{gathered} \sigma _{{xx}}^{p} \hfill \\ \sigma _{{yy}}^{p} \hfill \\ \sigma _{{xz}}^{p} \hfill \\ \sigma _{{yz}}^{p} \hfill \\ \sigma _{{xy}}^{p} \hfill \\ \end{gathered} \right\}=\left[ {\begin{array}{*{20}{c}} {{{\bar {C}}_{11}}}&{{{\bar {C}}_{12}}}&0&0&0 \\ {{{\bar {C}}_{12}}}&{{{\bar {C}}_{22}}}&0&0&0 \\ 0&0&{{{\bar {C}}_{55}}}&0&0 \\ 0&0&0&{{{\bar {C}}_{44}}}&0 \\ 0&0&0&0&{{{\bar {C}}_{66}}} \end{array}} \right]\left\{ \begin{gathered} {\varepsilon _{xx}} \\ {\varepsilon _{yy}} \\ {\gamma _{xz}} \\ {\gamma _{yz}} \\ {\gamma _{xy}} \\ \end{gathered} \right\} - \left[ {\begin{array}{*{20}{c}} 0&0&{{{\bar {e}}_{31}}} \\ 0&0&{{{\bar {e}}_{32}}} \\ {{{\bar {e}}_{15}}}&0&0 \\ 0&{{{\bar {e}}_{24}}}&0 \\ 0&0&0 \end{array}} \right]\left\{ \begin{gathered} {E_x} \hfill \\ {E_y} \hfill \\ {E_z} \hfill \\ \end{gathered} \right\}$$

3 $$\left\{ \begin{gathered} {D_x} \hfill \\ {D_y} \hfill \\ {D_z} \hfill \\ \end{gathered} \right\}=\left[ {\begin{array}{*{20}{c}} 0&0&{{{\bar {e}}_{15}}}&0&0 \\ 0&0&0&{{{\bar {e}}_{24}}}&0 \\ {{{\bar {e}}_{31}}}&{{{\bar {e}}_{32}}}&0&0&0 \end{array}} \right]\left\{ \begin{gathered} {\varepsilon _{xx}} \\ {\varepsilon _{yy}} \\ {\gamma _{xz}} \\ {\gamma _{yz}} \\ {\gamma _{xy}} \\ \end{gathered} \right\}+\left[ {\begin{array}{*{20}{c}} {{{\bar {s}}_{11}}}&0&0 \\ 0&{{{\bar {s}}_{22}}}&0 \\ 0&0&{{{\bar {s}}_{33}}} \end{array}} \right]\left\{ \begin{gathered} {E_x} \hfill \\ {E_y} \hfill \\ {E_z} \hfill \\ \end{gathered} \right\}$$

where ${\bar {e}_{ij}}$, ${\bar {s}_{ij}}$, and ${\bar {c}_{ij}}$ represent the piezoelectric, dielectric, and elastic coefficients, respectively. The surface constitutive equation for piezoelectric materials can be expressed as follows^55^:

4 $$\left\{ \begin{gathered} \sigma _{{xx}}^{{(S)}} \hfill \\ \sigma _{{yy}}^{{(S)}} \hfill \\ \sigma _{{xz}}^{{(S)}} \hfill \\ \sigma _{{yz}}^{{(S)}} \hfill \\ \sigma _{{xy}}^{{(S)}} \hfill \\ \end{gathered} \right\}=\left[ {\begin{array}{*{20}{c}} {{{\bar {C}}^{(S)}}_{{11}}}&{{{\bar {C}}^{(S)}}_{{12}}}&0&0&0 \\ {{{\bar {C}}^{(S)}}_{{12}}}&{{{\bar {C}}^{(S)}}_{{22}}}&0&0&0 \\ 0&0&0&0&0 \\ 0&0&0&0&0 \\ 0&0&0&0&{{{\bar {C}}^{(S)}}_{{66}}} \end{array}} \right]\left\{ \begin{gathered} {\varepsilon _{xx}} \\ {\varepsilon _{yy}} \\ {\gamma _{xz}} \\ {\gamma _{yz}} \\ {\gamma _{xy}} \\ \end{gathered} \right\} - \left[ {\begin{array}{*{20}{c}} 0&0&{{{\bar {e}}^{(S)}}_{{31}}} \\ 0&0&{{{\bar {e}}^{(S)}}_{{32}}} \\ 0&0&0 \\ 0&0&0 \\ 0&0&0 \end{array}} \right]\left\{ \begin{gathered} {E_x} \hfill \\ {E_y} \hfill \\ {E_z} \hfill \\ \end{gathered} \right\}+\left\{ \begin{gathered} 0 \\ 0 \\ {\sigma ^{(S)}}\frac{{\partial w}}{{\partial x}} \\ {\sigma ^{(S)}}\frac{{\partial w}}{{\partial x}} \\ 0 \\ \end{gathered} \right\}$$

5 $$\left\{ \begin{gathered} D_{x}^{{(S)}} \hfill \\ D_{y}^{{(S)}} \hfill \\ D_{z}^{{(S)}} \hfill \\ \end{gathered} \right\}=\left[ {\begin{array}{*{20}{c}} 0&0&{{{\bar {e}}^{(S)}}_{{15}}}&0&0 \\ 0&0&0&{{{\bar {e}}^{(S)}}_{{24}}}&0 \\ {{{\bar {e}}^{(S)}}_{{31}}}&{{{\bar {e}}^{(S)}}_{{32}}}&0&0&0 \end{array}} \right]\left\{ \begin{gathered} {\varepsilon _{xx}} \\ {\varepsilon _{yy}} \\ {\gamma _{xz}} \\ {\gamma _{yz}} \\ {\gamma _{xy}} \\ \end{gathered} \right\}+\left[ {\begin{array}{*{20}{c}} {\bar {s}_{{11}}^{{(S)}}}&0&0 \\ 0&{\bar {s}_{{22}}^{{(S)}}}&0 \\ 0&0&{\bar {s}_{{33}}^{{(S)}}} \end{array}} \right]\left\{ \begin{gathered} {E_x} \hfill \\ {E_y} \hfill \\ {E_z} \hfill \\ \end{gathered} \right\}$$

The surface piezoelectric coefficient, surface dielectric coefficient, surface elasticity coefficient, and surface stress are denoted by $\bar {e}_{{ij}}^{{(S)}}$, $\bar {s}_{{ij}}^{{(S)}}$, $\bar {c}_{{ij}}^{{(S)}}$, and ${\sigma ^{(S)}}$, respectively. The electric field and electric displacement are denoted by ${E_i}$ and ${D_i}$, respectively. The potential distribution is expressed as^56^

6 $${\bar {\varphi }_{ac}}=\sin \left[ {\frac{\pi }{{{h_p}}}\left( {z - \frac{{{h_c}}}{2}} \right)} \right]{\varphi _{ac}}+\frac{{z - \frac{{{h_c}}}{2}}}{{{h_p}}}{V_0},{\text{ }}{\bar {\varphi }_{se}}=\sin \left[ {\frac{\pi }{{{h_p}}}\left( { - z - \frac{{{h_c}}}{2}} \right)} \right]{\varphi _{se}}.$$

The space potential distribution of the piezoelectric layer is denoted by ${\varphi _{{\text{ac}}}}$, and ${\varphi _{se}}$. The electrical field is expressed as $E= - \nabla \bar {\varphi }$, where ${V_0}$ is the voltage.

According to the second-order shear deformation theory (SSDT), the displacement field is^54^

7 $$\begin{gathered} \bar {u}=u - z\frac{{\partial {w_b}}}{{\partial x}} - f(z)\frac{{\partial {w_s}}}{{\partial x}}, \hfill \\ \bar {v}=v - z\frac{{\partial {w_b}}}{{\partial y}} - f(z)\frac{{\partial {w_s}}}{{\partial y}}, \hfill \\ \bar {w}={w_b}+{w_s}, \hfill \\ \end{gathered}$$

where *u* and *v* are the lateral and longitudinal displacements, and${w_b}$and${w_s}$ are the bending and shear displacements, respectively. Moreover, $f(z)=\frac{h}{\pi }\sin {{\pi z} \mathord{\left/ {\vphantom {{\pi z} h}} \right. \kern-0pt} h}$ is the shear form function, and ${{df(z)} \mathord{\left/ {\vphantom {{df(z)} {dz}}} \right. \kern-0pt} {dz}}=1 - g(z)$. Therefore, the strain-displacement relationship is presented as follows^54^ :

8 $$\begin{gathered} {\varepsilon _{xx}}=\frac{{\partial u}}{{\partial x}} - z\frac{{{\partial ^2}{w_b}}}{{\partial {x^2}}} - f(z)\frac{{{\partial ^2}{w_s}}}{{\partial {x^2}}}, \hfill \\ {\varepsilon _{yy}}=\frac{{\partial v}}{{\partial y}} - z\frac{{{\partial ^2}{w_b}}}{{\partial {y^2}}} - f(z)\frac{{{\partial ^2}{w_s}}}{{\partial {y^2}}}, \hfill \\ {\gamma _{xz}}=g(z)\frac{{\partial {w_s}}}{{\partial x}}, \hfill \\ {\gamma _{yz}}=g(z)\frac{{\partial {w_s}}}{{\partial y}}, \hfill \\ {\gamma _{xy}}=\frac{{\partial u}}{{\partial y}}+\frac{{\partial v}}{{\partial x}} - z\left( {2\frac{{{\partial ^2}{w_b}}}{{\partial x\partial y}}} \right) - f(z)\left( {2\frac{{{\partial ^2}{w_s}}}{{\partial x\partial y}}} \right). \hfill \\ \end{gathered}$$

### Control equations

According to the Hamilton’s principle^55^

9 $$\int_{0}^{t} {\delta \left( {U - V+W} \right){\text{d}}t} =0,$$

The strain energy, kinetic energy, and work done by external forces are denoted by *U*, *V*, and *W*, respectively. In addition, the kinetic energy is expressed as^55^

10 $$\delta V=\int_{0}^{a} {\int_{0}^{b} {\left[ \begin{gathered} {I_0}\left[ {\frac{{\partial u}}{{\partial t}}\frac{{\partial \delta u}}{{\partial t}}+\frac{{\partial v}}{{\partial t}}\frac{{\partial \delta v}}{{\partial t}}+\left( {\frac{{\partial {w_b}}}{{\partial t}}+\frac{{\partial {w_s}}}{{\partial t}}} \right)\left( {\frac{{\partial \delta {w_b}}}{{\partial t}}+\frac{{\partial \delta {w_s}}}{{\partial t}}} \right)} \right] \hfill \\ - {I_1}\left( {\frac{{\partial u}}{{\partial t}}\frac{{{\partial ^2}\delta {w_b}}}{{\partial t\partial x}}+\frac{{\partial v}}{{\partial t}}\frac{{{\partial ^2}\delta {w_b}}}{{\partial t\partial y}}+\frac{{{\partial ^2}{w_b}}}{{\partial t\partial x}}\frac{{\partial \delta u}}{{\partial t}}+\frac{{{\partial ^2}{w_b}}}{{\partial t\partial y}}\frac{{\partial \delta v}}{{\partial t}}} \right) \hfill \\ +{I_2}\left( {\frac{{{\partial ^2}{w_b}}}{{\partial t\partial y}}\frac{{{\partial ^2}\delta {w_b}}}{{\partial t\partial y}}+\frac{{{\partial ^2}{w_b}}}{{\partial t\partial x}}\frac{{{\partial ^2}\delta {w_b}}}{{\partial t\partial x}}} \right)+{J_3}\left( {\frac{{{\partial ^2}{w_s}}}{{\partial t\partial y}}\frac{{{\partial ^2}\delta {w_s}}}{{\partial t\partial y}}+\frac{{{\partial ^2}{w_s}}}{{\partial t\partial x}}\frac{{{\partial ^2}\delta {w_s}}}{{\partial t\partial x}}} \right) \hfill \\ - {J_1}\left( {\frac{{\partial u}}{{\partial t}}\frac{{{\partial ^2}\delta {w_s}}}{{\partial t\partial x}}+\frac{{\partial v}}{{\partial t}}\frac{{{\partial ^2}\delta {w_s}}}{{\partial t\partial y}}+\frac{{{\partial ^2}{w_s}}}{{\partial t\partial x}}\frac{{\partial \delta u}}{{\partial t}}+\frac{{{\partial ^2}{w_s}}}{{\partial t\partial y}}\frac{{\partial \delta v}}{{\partial t}}} \right) \hfill \\ +{J_2}\left( {\frac{{{\partial ^2}{w_b}}}{{\partial t\partial x}}\frac{{{\partial ^2}\delta {w_s}}}{{\partial t\partial x}}+\frac{{{\partial ^2}{w_b}}}{{\partial t\partial y}}\frac{{{\partial ^2}\delta {w_s}}}{{\partial t\partial y}}+\frac{{{\partial ^2}{w_s}}}{{\partial t\partial x}}\frac{{{\partial ^2}\delta {w_b}}}{{\partial t\partial x}}+\frac{{{\partial ^2}{w_s}}}{{\partial t\partial y}}\frac{{{\partial ^2}\delta {w_b}}}{{\partial t\partial y}}} \right) \hfill \\ \end{gathered} \right]} } {\text{d}}y{\text{d}}x,$$.

The moment of inertia can be expressed as follows^55^:

11 $$\left( {{I_0},{I_1},{I_2},{J_3},{J_2},{J_1}} \right)=\left[ \begin{gathered} \int_{{ - {h_c}/2 - {h_p}}}^{{ - {h_c}/2}} {\left( {1,z,{z^2},{f^2},zf,f} \right)} {\rho _p}{\text{d}}z+{\rho _s}\left( {1,z,{z^2},{f^2},zf,f} \right)\left| {_{{z= - {h_c}/2 - {h_p}}}} \right. \hfill \\ +{\rho _s}\left( {1,z,{z^2},{f^2},zf,f} \right)\left| {_{{z= - {h_c}/2}}} \right.+\int_{{ - {h_c}/2}}^{{{h_c}/2}} {\left( {1,z,{z^2},{f^2},zf,f} \right)} {\rho _c}\left( z \right){\text{d}}z \hfill \\ +\int_{{{h_c}/2}}^{{{h_c}/2+{h_p}}} {\left( {1,z,{z^2},{f^2},zf,f} \right)} {\rho _p}{\text{d}}z+{\rho _s}\left( {1,z,{z^2},{f^2},zf,f} \right)\left| {_{{z={h_c}/2}}} \right. \hfill \\ +{\rho _s}\left( {1,z,{z^2},{f^2},zf,f} \right)\left| {_{{z={h_c}/2+{h_p}}}} \right. \hfill \\ \end{gathered} \right],$$

where ${\rho _{\text{s}}}$ and ${\rho _{\text{p}}}$ denote the surface density and bulk density of the piezoelectric material, respectively. ${\rho _{\text{e}}}$ denotes the density of the middle layer. The strain energy of the nanoplate is written as^54^

12 $$\begin{gathered} \delta U=\int_{0}^{a} {\int_{0}^{b} {\left[ \begin{gathered} {N_{xx}}\left( {\frac{{\partial \delta u}}{{\partial x}}} \right) - M_{{xx}}^{b}\left( {\frac{{{\partial ^2}\delta {w_b}}}{{\partial {x^2}}}} \right) - M_{{xx}}^{s}\left( {\frac{{{\partial ^2}\delta {w_s}}}{{\partial {x^2}}}} \right)+{N_{yy}}\left( {\frac{{\partial \delta v}}{{\partial y}}} \right) - M_{{yy}}^{b}\left( {\frac{{{\partial ^2}\delta {w_b}}}{{\partial {y^2}}}} \right) - M_{{yy}}^{s}\left( {\frac{{{\partial ^2}\delta {w_s}}}{{\partial {y^2}}}} \right)+ \hfill \\ {N_{xy}}\left[ {\left( {\frac{{\partial \delta u}}{{\partial y}}+\frac{{\partial \delta v}}{{\partial x}}} \right)} \right] - 2M_{{xy}}^{b}\left( {\frac{{{\partial ^2}\delta {w_b}}}{{\partial x\partial y}}} \right) - 2M_{{xy}}^{s}\left( {\frac{{{\partial ^2}\delta {w_s}}}{{\partial x\partial y}}} \right)+{Q_{xz}}\left( {\frac{{\partial \delta {w_s}}}{{\partial x}}} \right)+{Q_{yz}}\left( {\frac{{\partial \delta {w_s}}}{{\partial y}}} \right) \hfill \\ \end{gathered} \right]} } {\text{d}}y{\text{d}}x \hfill \\ {\text{ }}+\int {\int_{{ - {h_c}/2}}^{{{h_c}/2}} {\left( { - {D_x}\delta E_{x}^{{{\text{ac}}}} - {D_y}\delta E_{y}^{{{\text{ac}}}} - {D_z}\delta E_{z}^{{{\text{ac}}}}} \right){\text{dzd}}A} } \hfill \\ \end{gathered}$$

where the forces and moments are described as

13 $$\begin{gathered} \left( {{N_i},M_{i}^{b},M_{i}^{s}} \right)=\left( {\int_{{ - {h_c}/2 - {h_p}}}^{{ - {h_c}/2}} {\left( {1,z,f} \right)\sigma _{i}^{p}} dz+\left( {1,z,f} \right)\sigma _{i}^{s}\left| {_{{z= - {h_c}/2 - {h_p}}}} \right.+\left( {1,z,f} \right)\sigma _{i}^{s}\left| {_{{z= - {h_c}/2}}} \right.} \right)+\int_{{ - {h_c}/2}}^{{{h_c}/2}} {\left( {1,z,f} \right)\sigma _{i}^{c}} dz \hfill \\ {\text{ }}+\left( {\int_{{{h_c}/2}}^{{{h_c}/2+{h_p}}} {\left( {1,z,f} \right)\sigma _{i}^{p}} dz+\left( {1,z,f} \right)\sigma _{i}^{s}\left| {_{{z={h_c}/2}}} \right.+\left( {1,z,f} \right)\sigma _{i}^{s}\left| {_{{z={h_c}/2+{h_p}}}} \right.} \right){\text{ }}for{\text{ }}\left( {i=xx,yy,xy} \right). \hfill \\ \end{gathered}$$

14 $$\begin{gathered} {Q_i}=\left( {\int_{{ - {h_c}/2 - {h_p}}}^{{ - {h_c}/2}} {g\sigma _{i}^{p}} dz+g\sigma _{i}^{s}\left| {_{{z= - {h_c}/2 - {h_p}}}} \right.+g\sigma _{i}^{s}\left| {_{{z= - {h_c}/2}}} \right.} \right)+\int_{{ - {h_c}/2}}^{{{h_c}/2}} {g\sigma _{i}^{c}} dz \hfill \\ {\text{ }}+\left( {\int_{{{h_c}/2}}^{{{h_c}/2+{h_p}}} {g\sigma _{i}^{p}} dz+g\sigma _{i}^{s}\left| {_{{z={h_c}/2}}} \right.+g\sigma _{i}^{s}\left| {_{{z={h_c}/2+{h_p}}}} \right.} \right){\text{ for }}\left( {i=xz,yz} \right). \hfill \\ \end{gathered}$$

The external work is defined as follows^55^:

15 $$\delta W=\int_{0}^{a} {\int_{0}^{b} {\left\{ \begin{gathered} {k_w}\left( {{w_b}+{w_s}} \right)+{c_d}\left( {\frac{{\partial {w_b}}}{{\partial t}}+\frac{{\partial {w_s}}}{{\partial t}}} \right) - {k_p}\left[ {\frac{{{\partial ^2}\left( {{w_b}+{w_s}} \right)}}{{\partial {x^2}}}+\frac{{{\partial ^2}\left( {{w_b}+{w_s}} \right)}}{{\partial {y^2}}}} \right] \hfill \\ +N_{{xx}}^{V}\frac{{{\partial ^2}\left( {{w_b}+{w_s}} \right)}}{{\partial {x^2}}}+N_{{yy}}^{V}\frac{{{\partial ^2}\left( {{w_b}+{w_s}} \right)}}{{\partial {y^2}}} \hfill \\ \end{gathered} \right\}} } \left( {\delta {w_b}+\delta {w_s}} \right){\text{d}}y{\text{d}}x,$$

where the elasticity, shear, and damping coefficients are denoted by ${k_w}$, ${k_p}$, and ${c_d}$, respectively. Further, the voltage loads are indicated by $N_{x}^{{\text{E}}}$ and $N_{y}^{{\text{E}}}$.

16 $$N_{{xx}}^{V}=N_{{yy}}^{V}={\bar {e}_{31}}{V_0}.$$

The control equations are as follows:

17 $$\frac{{\partial \left( {{N_{xx}}+N_{{xx}}^{s}} \right)}}{{\partial x}}+\frac{{\partial \left( {{N_{xy}}+N_{{xy}}^{s}} \right)}}{{\partial y}}={I_0}\frac{{{\partial ^2}u}}{{\partial {t^2}}} - {I_1}\frac{{{\partial ^3}{w_b}}}{{\partial {t^2}\partial x}} - {J_1}\frac{{{\partial ^3}{w_s}}}{{\partial {t^2}\partial x}},$$

18 $$\frac{{\partial \left( {{N_{xy}}+N_{{xy}}^{s}} \right)}}{{\partial x}}+\frac{{\partial \left( {{N_{yy}}+N_{{yy}}^{s}} \right)}}{{\partial y}}={I_0}\frac{{{\partial ^2}v}}{{\partial {t^2}}} - {I_1}\frac{{{\partial ^3}{w_b}}}{{\partial {t^2}\partial y}} - {J_1}\frac{{{\partial ^3}{w_s}}}{{\partial {t^2}\partial y}},$$

19 $$\begin{gathered} \frac{{{\partial ^2}\left( {M_{{xx}}^{{bb}}+M_{{xx}}^{{bs}}} \right)}}{{\partial {x^2}}}+2\frac{{{\partial ^2}\left( {M_{{xy}}^{{bb}}+M_{{xy}}^{{bs}}} \right)}}{{\partial x\partial y}}+\frac{{{\partial ^2}\left( {M_{{yy}}^{{bb}}+M_{{yy}}^{{bs}}} \right)}}{{\partial {y^2}}} \hfill \\ +{k_p}{\nabla ^2}\left( {{w_b}+{w_s}} \right) - {k_w}\left( {{w_b}+{w_s}} \right) - {c_d}\left( {\frac{{\partial {w_b}}}{{\partial t}}+\frac{{\partial {w_s}}}{{\partial t}}} \right) - N_{{xx}}^{V}{\nabla ^2}\left( {{w_b}+{w_s}} \right) \hfill \\ ={I_0}\left( {\frac{{{\partial ^2}{w_b}}}{{\partial {t^2}}}+\frac{{{\partial ^2}{w_s}}}{{\partial {t^2}}}} \right)+{I_1}\left( {\frac{{{\partial ^3}u}}{{\partial {t^2}\partial x}}+\frac{{{\partial ^3}v}}{{\partial {t^2}\partial y}}} \right) - {I_2}{\nabla ^2}\frac{{{\partial ^2}{w_b}}}{{\partial {t^2}}} - {J_2}{\nabla ^2}\frac{{{\partial ^2}{w_s}}}{{\partial {t^2}}}, \hfill \\ \end{gathered}$$

20 $$\begin{gathered} \frac{{{\partial ^2}\left( {M_{{xx}}^{{sb}}+M_{{xx}}^{{ss}}} \right)}}{{\partial {x^2}}}+2\frac{{{\partial ^2}\left( {M_{{xy}}^{{sb}}+M_{{xy}}^{{ss}}} \right)}}{{\partial x\partial y}}+\frac{{{\partial ^2}\left( {M_{{yy}}^{{sb}}+M_{{yy}}^{{ss}}} \right)}}{{\partial {y^2}}}+\frac{{\partial \left( {{Q_{xz}}+Q_{{xz}}^{s}} \right)}}{{\partial x}}+\frac{{\partial \left( {{Q_{yz}}+Q_{{yz}}^{s}} \right)}}{{\partial y}} \hfill \\ +{k_p}{\nabla ^2}\left( {{w_b}+{w_s}} \right) - {k_w}\left( {{w_b}+{w_s}} \right) - {c_d}\left( {\frac{{\partial {w_b}}}{{\partial t}}+\frac{{\partial {w_s}}}{{\partial t}}} \right) - N_{{xx}}^{V}{\nabla ^2}\left( {{w_b}+{w_s}} \right) \hfill \\ ={I_0}\left( {\frac{{{\partial ^2}{w_b}}}{{\partial {t^2}}}+\frac{{{\partial ^2}{w_s}}}{{\partial {t^2}}}} \right)+{J_1}\left( {\frac{{{\partial ^3}u}}{{\partial {t^2}\partial x}}+\frac{{{\partial ^3}v}}{{\partial {t^2}\partial y}}} \right) - {J_2}{\nabla ^2}\frac{{{\partial ^2}{w_b}}}{{\partial {t^2}}} - {J_3}{\nabla ^2}\frac{{{\partial ^2}{w_s}}}{{\partial {t^2}}}, \hfill \\ \end{gathered}$$

21 $$\int\limits_{{ - {h_c}/2 - h/p}}^{{ - {h_c}/2}} {\left( {\frac{{\partial {D_x}}}{{\partial x}}\sin \left[ {\beta \left( { - z - \frac{{{h_{\text{c}}}}}{2}} \right)} \right]+\frac{{\partial {D_y}}}{{\partial x}}\sin \left[ {\beta \left( { - z - \frac{{{h_{\text{c}}}}}{2}} \right)} \right]+{D_z}\beta \cos \left( { - z - \frac{{{h_{\text{c}}}}}{2}} \right)} \right)} {\text{d}}z=0$$

22 $$\int\limits_{{{h_c}/2}}^{{{h_c}/2+h/p}} {\left( {\frac{{\partial {D_x}}}{{\partial x}}\sin \left[ {\beta \left( {z - \frac{{{h_{\text{c}}}}}{2}} \right)} \right]+\frac{{\partial {D_y}}}{{\partial x}}\sin \left[ {\beta \left( {z - \frac{{{h_{\text{c}}}}}{2}} \right)} \right] - {D_z}\beta \cos \left( {z - \frac{{{h_{\text{c}}}}}{2}} \right)} \right)} {\text{d}}z=0$$

### Equations of motion

The scale-dependent eigenstructure equations for piezoelectric materials are given as follows^30^:

23 $$\begin{gathered} \left( {1 - {\mu _1}{\nabla ^2}} \right)\left( {1 - {\mu _0}{\nabla ^2}} \right){\sigma _{ij}}=\left[ {\left( {1 - {\mu _1}{\nabla ^2}} \right) - \eta \left( {1 - {\mu _0}{\nabla ^2}} \right){\nabla ^2}} \right]\left( {{c_{ijkl}}{\varepsilon _{kl}} - {e_{mij}}{E_m}} \right), \hfill \\ \left( {1 - {\mu _1}{\nabla ^2}} \right)\left( {1 - {\mu _0}{\nabla ^2}} \right){D_i}=\left[ {\left( {1 - {\mu _1}{\nabla ^2}} \right) - \eta \left( {1 - {\mu _0}{\nabla ^2}} \right){\nabla ^2}} \right]\left( {{e_{ikl}}{\varepsilon _{kl}}+{s_{im}}{E_m}} \right), \hfill \\ \end{gathered}$$

where $\eta ={l^2}$ and $\mu ={\left( {{e_0}a} \right)^2}$ indicate the LSP and NP, respectively; $\Gamma =1 - \mu {\nabla ^2},\;\Upsilon =1 - \eta {\nabla ^2}$. The moment equation is expressed as

24 $$\begin{gathered} \Gamma \left( \begin{gathered} N_{{xx}}^{b} \hfill \\ N_{{yy}}^{b} \hfill \\ N_{{xy}}^{b} \hfill \\ \end{gathered} \right)=\Upsilon \left\{ \begin{gathered} \left[ {\begin{array}{*{20}{c}} {{A_{11}}}&{{A_{12}}}&0 \\ {{A_{12}}}&{{A_{22}}}&0 \\ 0&0&{{A_{66}}} \end{array}} \right]\left( \begin{gathered} \frac{{\partial u}}{{\partial x}} \\ \frac{{\partial v}}{{\partial y}} \\ \frac{{\partial u}}{{\partial y}}+\frac{{\partial v}}{{\partial x}} \\ \end{gathered} \right)+\left[ {\begin{array}{*{20}{c}} {{B_{11}}}&{{B_{12}}}&0 \\ {{B_{12}}}&{{B_{22}}}&0 \\ 0&0&{{B_{66}}} \end{array}} \right]\left( \begin{gathered} - \frac{{{\partial ^2}{w_b}}}{{\partial {x^2}}} \\ - \frac{{{\partial ^2}{w_b}}}{{\partial {y^2}}} \\ - 2\frac{{{\partial ^2}{w_b}}}{{\partial x\partial y}} \\ \end{gathered} \right) \hfill \\ +\left[ {\begin{array}{*{20}{c}} {{R_{11}}}&{{R_{12}}}&0 \\ {{R_{12}}}&{{R_{22}}}&0 \\ 0&0&{{R_{66}}} \end{array}} \right]\left( \begin{gathered} - \frac{{{\partial ^2}{w_s}}}{{\partial {x^2}}} \\ - \frac{{{\partial ^2}{w_s}}}{{\partial {y^2}}} \\ - 2\frac{{{\partial ^2}{w_s}}}{{\partial x\partial y}} \\ \end{gathered} \right)+\left( \begin{gathered} N_{{xx}}^{{bV}} \\ N_{{yy}}^{{bV}} \\ 0 \\ \end{gathered} \right) \hfill \\ \end{gathered} \right\}, \hfill \\ \Gamma \left( \begin{gathered} Q_{{xz}}^{b} \hfill \\ Q_{{yz}}^{b} \hfill \\ \end{gathered} \right)=\Upsilon \left\{ {\left[ {\begin{array}{*{20}{c}} {{T_{55}}}&0 \\ 0&{{T_{44}}} \end{array}} \right]\left( \begin{gathered} \frac{{\partial {w_s}}}{{\partial x}} \\ \frac{{\partial {w_s}}}{{\partial y}} \\ \end{gathered} \right)+\left[ {\begin{array}{*{20}{c}} {E_{{15}}^{{ac}}}&0 \\ 0&{E_{{24}}^{{ac}}} \end{array}} \right]\left( \begin{gathered} \frac{{\partial {{\bar {\varphi }}_{ac}}}}{{\partial x}} \\ \frac{{\partial {{\bar {\varphi }}_{ac}}}}{{\partial y}} \\ \end{gathered} \right)} \right\}. \hfill \\ \end{gathered}$$

25 $$\Gamma \left( \begin{gathered} M_{{xx}}^{{bb}} \hfill \\ M_{{yy}}^{{bb}} \hfill \\ M_{{xy}}^{{bb}} \hfill \\ \end{gathered} \right)=\Upsilon \left\{ \begin{gathered} \left[ {\begin{array}{*{20}{c}} {{B_{11}}}&{{B_{12}}}&0 \\ {{B_{12}}}&{{B_{22}}}&0 \\ 0&0&{{B_{66}}} \end{array}} \right]\left( \begin{gathered} \frac{{\partial u}}{{\partial x}} \\ \frac{{\partial v}}{{\partial y}} \\ \frac{{\partial u}}{{\partial y}}+\frac{{\partial v}}{{\partial x}} \\ \end{gathered} \right)+\left[ {\begin{array}{*{20}{c}} {{D_{11}}}&{{D_{12}}}&0 \\ {{D_{12}}}&{{D_{22}}}&0 \\ 0&0&{{D_{66}}} \end{array}} \right]\left( \begin{gathered} - \frac{{{\partial ^2}{w_b}}}{{\partial {x^2}}} \\ - \frac{{{\partial ^2}{w_b}}}{{\partial {y^2}}} \\ - 2\frac{{{\partial ^2}{w_b}}}{{\partial x\partial y}} \\ \end{gathered} \right)+\left[ {\begin{array}{*{20}{c}} {{G_{11}}}&{{G_{12}}}&0 \\ {{G_{12}}}&{{G_{22}}}&0 \\ 0&0&{{G_{66}}} \end{array}} \right]\left( \begin{gathered} - \frac{{{\partial ^2}{w_s}}}{{\partial {x^2}}} \\ - \frac{{{\partial ^2}{w_s}}}{{\partial {y^2}}} \\ - 2\frac{{{\partial ^2}{w_s}}}{{\partial x\partial y}} \\ \end{gathered} \right) \hfill \\ +\left[ {\begin{array}{*{20}{c}} {E_{{31M_{{xx}}^{{bb}}}}^{a}}&0 \\ {E_{{32M_{{yy}}^{{bb}}}}^{a}}&0 \\ 0&0 \end{array}} \right]\left( \begin{gathered} {{\bar {\varphi }}_{ac}} \\ 0 \\ \end{gathered} \right)+\left( \begin{gathered} E_{{31M_{{xx}}^{{bb}}}}^{V} \\ E_{{32M_{{yy}}^{{bb}}}}^{V} \\ 0 \\ \end{gathered} \right) \hfill \\ \end{gathered} \right\}.$$

26 $$\Gamma \left( \begin{gathered} M_{{xx}}^{{sb}} \hfill \\ M_{{yy}}^{{sb}} \hfill \\ M_{{xy}}^{{sb}} \hfill \\ \end{gathered} \right)=\Upsilon \left\{ \begin{gathered} \left[ {\begin{array}{*{20}{c}} {{R_{11}}}&{{R_{12}}}&0 \\ {{R_{12}}}&{{R_{22}}}&0 \\ 0&0&{{R_{66}}} \end{array}} \right]\left( \begin{gathered} \frac{{\partial u}}{{\partial x}} \\ \frac{{\partial v}}{{\partial y}} \\ \frac{{\partial u}}{{\partial y}}+\frac{{\partial v}}{{\partial x}} \\ \end{gathered} \right)+\left[ {\begin{array}{*{20}{c}} {{G_{11}}}&{{G_{12}}}&0 \\ {{G_{12}}}&{{G_{22}}}&0 \\ 0&0&{{G_{66}}} \end{array}} \right]\left( \begin{gathered} - \frac{{{\partial ^2}{w_b}}}{{\partial {x^2}}} \\ - \frac{{{\partial ^2}{w_b}}}{{\partial {y^2}}} \\ - 2\frac{{{\partial ^2}{w_b}}}{{\partial x\partial y}} \\ \end{gathered} \right)+\left[ {\begin{array}{*{20}{c}} {{H_{11}}}&{{H_{12}}}&0 \\ {{H_{12}}}&{{H_{22}}}&0 \\ 0&0&{{H_{66}}} \end{array}} \right]\left( \begin{gathered} - \frac{{{\partial ^2}{w_s}}}{{\partial {x^2}}} \\ - \frac{{{\partial ^2}{w_s}}}{{\partial {y^2}}} \\ - 2\frac{{{\partial ^2}{w_s}}}{{\partial x\partial y}} \\ \end{gathered} \right) \hfill \\ +\left[ {\begin{array}{*{20}{c}} {E_{{31M_{{xx}}^{{sb}}}}^{a}}&0 \\ {E_{{32M_{{yy}}^{{sb}}}}^{a}}&0 \\ 0&0 \end{array}} \right]\left( \begin{gathered} {{\bar {\varphi }}_{ac}} \\ 0 \\ \end{gathered} \right)+\left( \begin{gathered} E_{{31M_{{xx}}^{{sb}}}}^{V} \\ E_{{32M_{{yy}}^{{sb}}}}^{V} \\ 0 \\ \end{gathered} \right) \hfill \\ \end{gathered} \right\}.$$

27 $$\begin{gathered} \Gamma \int_{{ - {h_c}/2 - {h_p}}}^{{ - {h_c}/2}} {\left\{ {\left( \begin{gathered} - \frac{{\partial {D_x}}}{{\partial x}} \hfill \\ - \frac{{\partial {D_y}}}{{\partial y}} \hfill \\ \end{gathered} \right)\sin \left[ {\beta \left( { - z - \frac{{{h_c}}}{2}} \right)} \right]} \right\}dz} =\Upsilon \left[ {\begin{array}{*{20}{c}} {F_{{15}}^{{se}}}&{F_{{11}}^{{se}}}&0&0 \\ 0&0&{F_{{24}}^{{se}}}&{F_{{22}}^{{se}}} \end{array}} \right]\left( \begin{gathered} \frac{{{\partial ^2}{w_s}}}{{\partial {x^2}}} \hfill \\ \frac{{{\partial ^2}{{\bar {\varphi }}_{se}}}}{{\partial {x^2}}} \hfill \\ \frac{{{\partial ^2}{w_s}}}{{\partial {y^2}}} \hfill \\ \frac{{{\partial ^2}{{\bar {\varphi }}_{se}}}}{{\partial {y^2}}} \hfill \\ \end{gathered} \right), \hfill \\ \Gamma \int_{{ - {h_c}/2 - {h_p}}}^{{ - {h_c}/2}} { - {D_z}\beta \cos \left[ {\beta \left( { - z - \frac{{{h_c}}}{2}} \right)} \right]dz} =\Upsilon \left( {H_{{31}}^{{se}}\frac{{{\partial ^2}{w_b}}}{{\partial {x^2}}}+K_{{31}}^{{se}}\frac{{{\partial ^2}{w_s}}}{{\partial {x^2}}}+H_{{32}}^{{se}}\frac{{{\partial ^2}{w_b}}}{{\partial {y^2}}}+K_{{32}}^{{se}}\frac{{{\partial ^2}{w_s}}}{{\partial {y^2}}}+F_{{33}}^{{se}}{{\bar {\varphi }}_{se}}} \right). \hfill \\ \end{gathered}$$

28 $$\begin{gathered} \Gamma \int_{{{h_c}/2}}^{{{h_c}/2+{h_p}}} {\left( \begin{gathered} - \frac{{\partial {D_x}}}{{\partial x}} \hfill \\ - \frac{{\partial {D_y}}}{{\partial y}} \hfill \\ \end{gathered} \right)\sin \left[ {\beta \left( {z - \frac{{{h_c}}}{2}} \right)} \right]dz} =\Upsilon \left[ {\begin{array}{*{20}{c}} {F_{{15}}^{{{\text{ac}}}}}&{F_{{11}}^{{{\text{ac}}}}}&0&0 \\ 0&0&{F_{{24}}^{{{\text{ac}}}}}&{F_{{22}}^{{{\text{ac}}}}} \end{array}} \right]\left( \begin{gathered} \frac{{{\partial ^2}{w_s}}}{{\partial {x^2}}} \hfill \\ \frac{{{\partial ^2}{{\bar {\varphi }}_{{\text{ac}}}}}}{{\partial {x^2}}} \hfill \\ \frac{{{\partial ^2}{w_s}}}{{\partial {y^2}}} \hfill \\ \frac{{{\partial ^2}{{\bar {\varphi }}_{{\text{ac}}}}}}{{\partial {y^2}}} \hfill \\ \end{gathered} \right), \hfill \\ \Gamma \int_{{{h_c}/2}}^{{{h_c}/2+{h_p}}} {{D_z}\beta \cos \left[ {\beta \left( {z - \frac{{{h_c}}}{2}} \right)} \right]dz} =\Upsilon \left( {H_{{31}}^{{{\text{ac}}}}\frac{{{\partial ^2}{w_b}}}{{\partial {x^2}}}+K_{{31}}^{{{\text{ac}}}}\frac{{{\partial ^2}{w_s}}}{{\partial {x^2}}}+H_{{32}}^{{{\text{ac}}}}\frac{{{\partial ^2}{w_b}}}{{\partial {y^2}}}+K_{{32}}^{{{\text{ac}}}}\frac{{{\partial ^2}{w_s}}}{{\partial {y^2}}}+F_{{33}}^{{{\text{ac}}}}{{\bar {\varphi }}_{{\text{ac}}}}} \right). \hfill \\ \end{gathered}$$

Combining Eqs. (24–28) and Eqs. (17–22), the equation of motion is obtained as follows:

29 $$\begin{gathered} \Upsilon \left\{ \begin{gathered} \left( {{A_{11}}+A_{{11}}^{s}} \right)\frac{{{\partial ^2}u}}{{\partial {x^2}}}{\text{+}}\left( {{A_{66}}+A_{{66}}^{s}} \right)\frac{{{\partial ^2}u}}{{\partial {y^2}}}+\left[ {\left( {{A_{12}}+{A_{66}}} \right)+\left( {A_{{12}}^{s}+A_{{66}}^{s}} \right)} \right]\frac{{{\partial ^2}v}}{{\partial x\partial y}} - \left( {{B_{11}}+B_{{11}}^{s}} \right)\frac{{{\partial ^3}{w_b}}}{{\partial {x^3}}} \hfill \\ - \left[ {\left( {{B_{12}}{\text{+}}2{B_{66}}} \right)+\left( {B_{{12}}^{s}+2B_{{66}}^{s}} \right)} \right]\frac{{{\partial ^3}{w_b}}}{{\partial x\partial {y^2}}} - \left( {{R_{11}}+R_{{11}}^{s}} \right)\frac{{{\partial ^3}{w_s}}}{{\partial {x^3}}} - \left[ {\left( {{R_{12}}+2{R_{66}}} \right)+\left( {R_{{12}}^{s}+2R_{{66}}^{s}} \right)} \right]\frac{{{\partial ^3}{w_s}}}{{\partial x\partial {y^2}}} \hfill \\ \end{gathered} \right\} \hfill \\ =\Gamma \left( {{I_0}\frac{{{\partial ^2}u}}{{\partial {t^2}}} - {I_1}\frac{{{\partial ^3}{w_b}}}{{\partial {t^2}\partial x}} - {J_1}\frac{{{\partial ^2}{w_s}}}{{\partial {t^2}\partial x}}} \right), \hfill \\ \end{gathered}$$

30 $$\begin{gathered} \Upsilon \left\{ \begin{gathered} \left[ {\left( {{A_{12}}+{A_{66}}} \right){\text{+}}\left( {A_{{12}}^{s}{\text{+}}A_{{66}}^{s}} \right)} \right]\frac{{{\partial ^2}u}}{{\partial x\partial y}}+\left( {{A_{66}}{\text{+}}A_{{66}}^{s}} \right)\frac{{{\partial ^2}v}}{{\partial {x^2}}}+\left( {{A_{22}}+A_{{22}}^{s}} \right)\frac{{{\partial ^2}v}}{{\partial {y^2}}} - \left( {{B_{22}}{\text{+}}B_{{22}}^{s}} \right)\frac{{{\partial ^3}{w_b}}}{{\partial {y^3}}} \hfill \\ - \left[ {\left( {{B_{12}}+2{B_{66}}} \right){\text{+}}\left( {B_{{12}}^{s}+2B_{{66}}^{s}} \right)} \right]\frac{{{\partial ^3}{w_b}}}{{\partial {x^2}\partial y}} - \left( {{R_{22}}{\text{+}}R_{{22}}^{s}} \right)\frac{{{\partial ^3}{w_s}}}{{\partial {y^3}}} - \left[ {\left( {{R_{12}}+2{R_{66}}} \right){\text{+}}\left( {R_{{12}}^{s}+2R_{{66}}^{s}} \right)} \right]\frac{{{\partial ^3}{w_s}}}{{\partial {x^2}\partial y}} \hfill \\ \end{gathered} \right\} \hfill \\ {\text{=}}\Gamma \left( {{I_0}\frac{{{\partial ^2}v}}{{\partial {t^2}}} - {I_1}\frac{{{\partial ^3}{w_b}}}{{\partial {t^2}\partial y}} - {J_1}\frac{{{\partial ^3}{w_s}}}{{\partial {t^2}\partial y}}} \right), \hfill \\ \end{gathered}$$

31 $$\begin{gathered} \Upsilon \left\{ \begin{gathered} \left( {{B_{11}}+B_{{11}}^{s}} \right)\frac{{{\partial ^3}u}}{{\partial {x^3}}}{\text{+}}\left[ {\left( {{B_{12}}{\text{+}}2{B_{66}}} \right){\text{+}}\left( {B_{{12}}^{s}{\text{+}}2B_{{_{{66}}}}^{s}} \right)} \right]\frac{{{\partial ^3}u}}{{\partial x\partial {y^2}}} \hfill \\ +\left( {{B_{22}}+B_{{22}}^{s}} \right)\frac{{{\partial ^3}v}}{{\partial {y^3}}}+\left[ {\left( {{B_{12}}+2{B_{66}}} \right){\text{+}}\left( {B_{{12}}^{s}+2B_{{_{{66}}}}^{s}} \right)} \right]\frac{{{\partial ^3}v}}{{\partial {x^2}\partial y}} \hfill \\ - \left( {{D_{11}}+D_{{11}}^{s}} \right)\frac{{{\partial ^4}{w_b}}}{{\partial {x^4}}} - 2\left[ {\left( {{D_{12}}+2{D_{66}}} \right)+\left( {D_{{12}}^{s}{\text{+}}2D_{{_{{66}}}}^{s}} \right)} \right]\frac{{{\partial ^4}{w_b}}}{{\partial {x^2}\partial {y^2}}} - \left( {{D_{22}}+D_{{22}}^{s}} \right)\frac{{{\partial ^4}{w_b}}}{{\partial {y^4}}} \hfill \\ - \left( {{G_{11}}+G_{{11}}^{s}} \right)\frac{{{\partial ^4}{w_s}}}{{\partial {x^4}}} - 2\left[ {\left( {{G_{12}}+2{G_{66}}} \right)+\left( {G_{{12}}^{s}{\text{+}}2G_{{_{{66}}}}^{s}} \right)} \right]\frac{{{\partial ^4}{w_s}}}{{\partial {x^2}\partial {y^2}}} - \left( {{G_{22}}+G_{{22}}^{s}} \right)\frac{{{\partial ^4}{w_s}}}{{\partial {y^4}}} \hfill \\ +E_{{31M_{{xx}}^{{bb}}}}^{s}{\nabla ^2}{{\bar {\varphi }}_s}+E_{{31M_{{xx}}^{{bb}}}}^{a}{\nabla ^2}{{\bar {\varphi }}_a}+E_{{31M_{{xx}}^{{bs}}}}^{s}{\nabla ^2}{{\bar {\varphi }}_s}+E_{{31M_{{xx}}^{{bs}}}}^{a}{\nabla ^2}{{\bar {\varphi }}_a} \hfill \\ \end{gathered} \right\} \hfill \\ +\Gamma \left\{ {{k_p}{\nabla ^2}\left( {{w_b}+{w_s}} \right) - {k_w}\left( {{w_b}+{w_s}} \right) - {c_d}\left( {\frac{{\partial {w_b}}}{{\partial t}}+\frac{{\partial {w_s}}}{{\partial t}}} \right) - N_{{xx}}^{V}{\nabla ^2}\left( {{w_b}+{w_s}} \right)} \right\} \hfill \\ =\Gamma \left[ {{I_0}\left( {\frac{{{\partial ^2}{w_b}}}{{\partial {t^2}}}+\frac{{{\partial ^2}{w_s}}}{{\partial {t^2}}}} \right)+{I_1}\left( {\frac{{{\partial ^3}u}}{{\partial {t^2}\partial x}}+\frac{{{\partial ^3}v}}{{\partial {t^2}\partial y}}} \right) - {I_2}{\nabla ^2}\frac{{{\partial ^2}{w_b}}}{{\partial {t^2}}} - {J_2}{\nabla ^2}\frac{{{\partial ^2}{w_s}}}{{\partial {t^2}}}} \right], \hfill \\ \end{gathered}$$

32 $$\begin{gathered} \Upsilon \left\{ \begin{gathered} \left( {{R_{11}}{\text{+}}R_{{11}}^{s}} \right)\frac{{{\partial ^3}u}}{{\partial {x^3}}}+\left[ {\left( {{R_{12}}+2{R_{66}}} \right)+\left( {R_{{12}}^{s}+2R_{{66}}^{s}} \right)} \right]\frac{{{\partial ^3}u}}{{\partial x\partial {y^2}}} \hfill \\ +\left( {{R_{22}}{\text{+}}R_{{22}}^{s}} \right)\frac{{{\partial ^3}v}}{{\partial {y^3}}}+\left[ {\left( {{R_{12}}+2{R_{66}}} \right){\text{+}}\left( {R_{{12}}^{s}+2R_{{66}}^{s}} \right)} \right]\frac{{{\partial ^3}v}}{{\partial {x^2}\partial y}} \hfill \\ - \left( {{G_{11}}{\text{+}}G_{{11}}^{s}} \right)\frac{{{\partial ^4}{w_b}}}{{\partial {x^4}}} - 2\left[ {\left( {{G_{12}}+2{G_{66}}} \right){\text{+}}\left( {G_{{12}}^{s}+2G_{{66}}^{s}} \right)} \right]\frac{{{\partial ^4}{w_b}}}{{\partial {x^2}\partial {y^2}}} - \left( {{G_{22}}{\text{+}}G_{{22}}^{s}} \right)\frac{{{\partial ^4}{w_b}}}{{\partial {y^4}}}+g_{\sigma }^{s}{\nabla ^2}{w_b} \hfill \\ - \left( {{H_{11}}{\text{+}}H_{{11}}^{s}} \right)\frac{{{\partial ^4}{w_s}}}{{\partial {x^4}}} - 2\left[ {\left( {{H_{12}}+2{H_{66}}} \right){\text{+}}\left( {H_{{12}}^{s}+2H_{{66}}^{s}} \right)} \right]\frac{{{\partial ^4}{w_s}}}{{\partial {x^2}\partial {y^2}}} - \left( {{H_{22}}{\text{+}}H_{{22}}^{s}} \right)\frac{{{\partial ^4}{w_s}}}{{\partial {y^4}}}{\text{+}}g_{\sigma }^{s}{\nabla ^2}{w_s}+{T_{55}}\frac{{{\partial ^2}{w_s}}}{{\partial {x^2}}}+{T_{44}}\frac{{{\partial ^2}{w_s}}}{{\partial {y^2}}} \hfill \\ +\left[ {\left( {E_{{31M_{{xx}}^{{sb}}}}^{s}+E_{{15}}^{s}} \right)+E_{{31M_{{xx}}^{{{\text{ss}}}}}}^{s}} \right]{\nabla ^2}{{\bar {\varphi }}_s}+\left[ {\left( {E_{{31M_{{xx}}^{{sb}}}}^{a}+E_{{15}}^{a}} \right)+E_{{31M_{{xx}}^{{{\text{ss}}}}}}^{a}} \right]{\nabla ^2}{{\bar {\varphi }}_a} \hfill \\ \end{gathered} \right\} \hfill \\ +\Gamma \left\{ {{k_p}{\nabla ^2}\left( {{w_b}+{w_s}} \right) - {k_w}\left( {{w_b}+{w_s}} \right) - {c_d}\left( {\frac{{\partial {w_b}}}{{\partial t}}+\frac{{\partial {w_s}}}{{\partial t}}} \right) - N_{{xx}}^{V}{\nabla ^2}\left( {{w_b}+{w_s}} \right)} \right\} \hfill \\ =\Gamma \left[ {{I_0}\left( {\frac{{{\partial ^2}{w_b}}}{{\partial {t^2}}}+\frac{{{\partial ^2}{w_s}}}{{\partial {t^2}}}} \right)+{J_1}\left( {\frac{{{\partial ^3}u}}{{\partial {t^2}\partial x}}+\frac{{{\partial ^3}v}}{{\partial {t^2}\partial y}}} \right) - {J_2}{\nabla ^2}\frac{{{\partial ^2}{w_b}}}{{\partial {t^2}}} - {J_3}{\nabla ^2}\frac{{{\partial ^2}{w_s}}}{{\partial {t^2}}}} \right], \hfill \\ \end{gathered}$$

33 $$\Xi =\left( {\begin{array}{*{20}{c}} {\hat {H}_{{31}}^{{se}}\frac{{{\partial ^2}{w_b}}}{{\partial {x^2}}}+\hat {H}_{{32}}^{{se}}\frac{{{\partial ^2}{w_b}}}{{\partial {y^2}}}+\left( {\hat {K}_{{31}}^{{{\text{se}}}}{\text{+}}\hat {F}_{{15}}^{{{\text{se}}}}} \right)\frac{{{\partial ^2}{w_s}}}{{\partial {x^2}}}} \\ {+\left( {\hat {K}_{{32}}^{{{\text{se}}}}{\text{+}}\hat {F}_{{24}}^{{{\text{se}}}}} \right)\frac{{{\partial ^2}{w_s}}}{{\partial {y^2}}}+\hat {F}_{{11}}^{{{\text{se}}}}\frac{{{\partial ^2}{{\bar {\varphi }}_{{\text{se}}}}}}{{\partial {x^2}}}{\text{+}}\hat {F}_{{22}}^{{{\text{se}}}}\frac{{{\partial ^2}{{\bar {\varphi }}_{{\text{se}}}}}}{{\partial {y^2}}}+\hat {F}_{{33}}^{{{\text{se}}}}{{\bar {\varphi }}_{{\text{se}}}}} \end{array}} \right){\text{=}}0.$$

34 $$\Upsilon \left( \begin{gathered} H_{{31}}^{{{\text{ac}}}}\frac{{{\partial ^2}{w_b}}}{{\partial {x^2}}}+H_{{32}}^{{{\text{ac}}}}\frac{{{\partial ^2}{w_b}}}{{\partial {y^2}}}+\left( {K_{{31}}^{{{\text{ac}}}}{\text{+}}F_{{15}}^{{{\text{ac}}}}} \right)\frac{{{\partial ^2}{w_s}}}{{\partial {x^2}}} \hfill \\ +\left( {K_{{32}}^{{{\text{ac}}}}{\text{+}}F_{{24}}^{{{\text{ac}}}}} \right)\frac{{{\partial ^2}{w_s}}}{{\partial {y^2}}}+F_{{11}}^{{{\text{ac}}}}\frac{{{\partial ^2}{{\bar {\varphi }}_{{\text{ac}}}}}}{{\partial {x^2}}}{\text{+}}F_{{22}}^{{{\text{ac}}}}\frac{{{\partial ^2}{{\bar {\varphi }}_{{\text{ac}}}}}}{{\partial {y^2}}}+F_{{33}}^{{{\text{ac}}}}{{\bar {\varphi }}_{{\text{ac}}}} \hfill \\ \end{gathered} \right){\text{=}}0.$$

### Eigenvalue equation

The displacement field is given by the following harmonic solution^53^:

35 $${\left[ {u,v,{w_b},{w_s},{{\bar {\varphi }}_{se}},{{\bar {\varphi }}_{ac}}} \right]^{\text{T}}}={{\mathbf{d}}_{\mathbf{0}}}{\text{e}^{\text{i}\left( {{k_1}x+{k_2}y - \omega t} \right)}},{{\mathbf{d}}_{\mathbf{0}}}={\left[ {{u_0},{v_0},{w_{b0}},{w_{s0}},{{\bar {\varphi }}_{se0}},{{\bar {\varphi }}_{ac0}}} \right]^{\text{T}}}.$$

Here, ${{\mathbf{d}}_{\mathbf{0}}}$ is the universal displacement constant, and *ω* is the angular frequency. *k*_1_ and *k*_2_ denote the WN in the *x* and *y* directions, respectively. The eigenvalue equation can be obtained by inserting Eq. (35) into Eqs. (29)–(34) as follows:

36 $$\left( {{\mathbf{{\rm K}}}+{\text{i}}{\mathbf{C}}\omega - {\mathbf{M}}{\omega ^2}} \right){{\mathbf{d}}_{\mathbf{0}}}={\mathbf{0}}.$$

The dispersion relation can be derived by solving the above equations.

## Results and discussion

The metallic layer material was made up of molybdenum (Mo) with the following material parameters: *E*_*e*_ = 329 × 10^9^, *p*_e_ = 10,280, and *v*_*e*_ = 0.34. Polyvinylidene fluoride (PVDF) was chosen as the piezoelectric material, and its material parameters can be found in Ref^55^. The surface parameters of the piezoelectric material are as follows: *C*_*ij_s*_ = *C*_*ij*_×*P*_1_, *e*_*ij_s*_ = *e*_*ij*_×*P*_2_, and *s*_*ij_s*_ = *s*_*ij*_×*P*_3_.

### Model validation

Currently, it is extremely difficult to conduct experiments and molecular dynamics simulations of piezoelectric sandwich nanoplates.

**Fig. 2:**
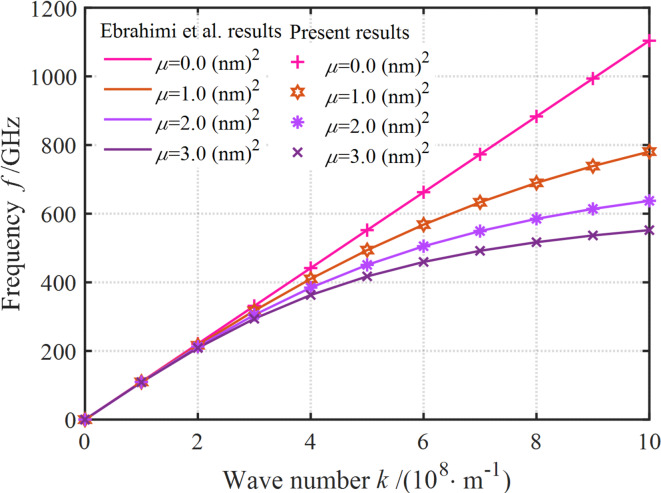
Comparison between our and reported frequency results for nanoplate.

Therefore, the results obtained in this study are validated through comparison with the dispersion properties of piezoelectric nanoplates or nanoplates. The structure is used for comparison when the upper and lower surface piezoelectric layers are not considered, the findings of this study simplify to a comparative analysis of wave propagation properties in configurations limited to a single piezoelectric layer. As shown in Fig. 2, the study of Ebrahimi et al.^30^ suggest that our results can fit well with the existing literature results, which indicates the accuracy of scale-dependent modeling.

### Wave propagation analysis

**Fig. 3:**
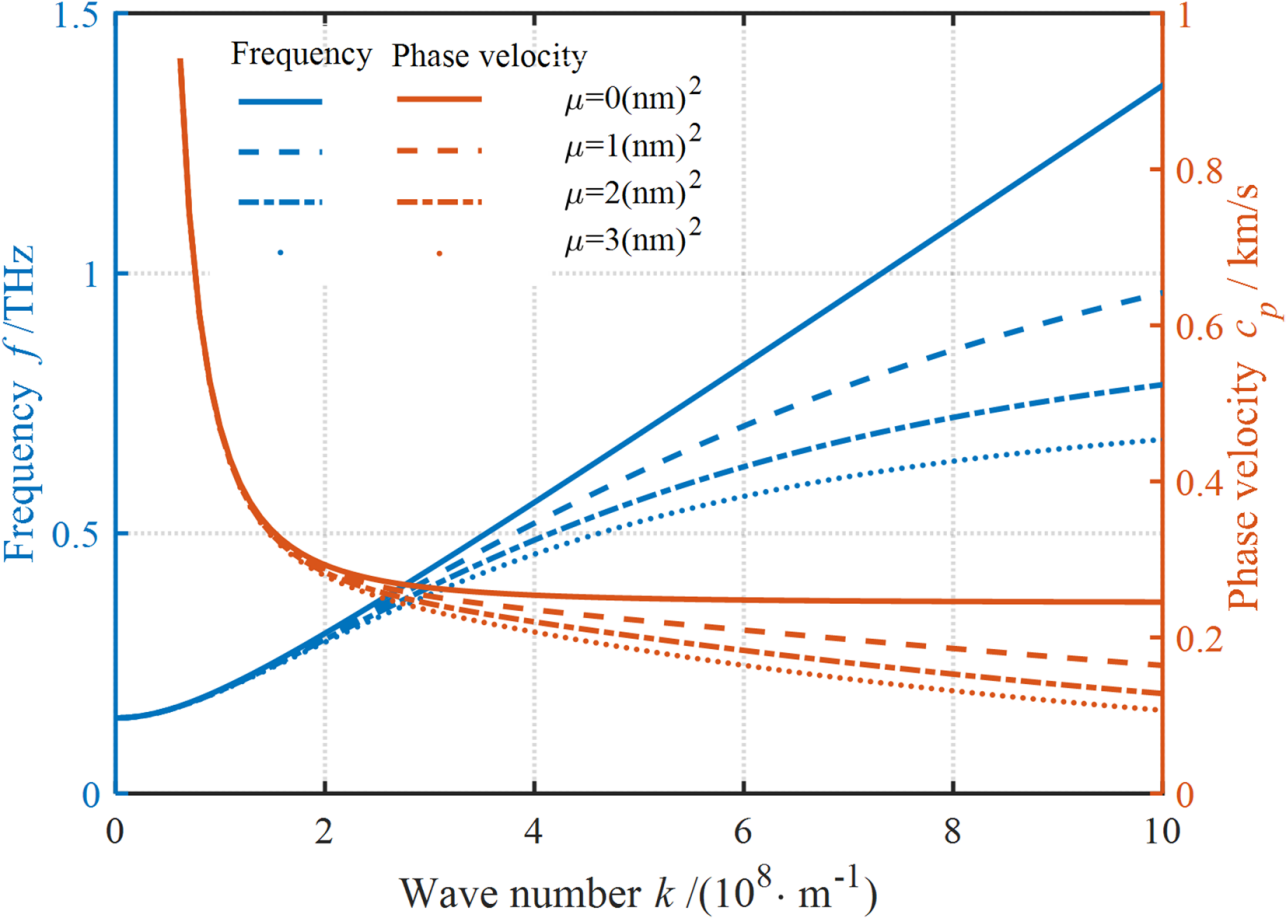
Impact of nonlocal stress fields on the dispersion curves.

It can be seen in Fig. 3 that the dispersion curves are affected by nonlocal stress fields. The results show that neglecting NP can degrade the existing model to the traditional localized model, i.e., there is a point in the structure where the stress only depends on its strain. Further, it is evident that the frequency is almost linearly proportional to the WN, while the PV first decreases sharply with the increase in WN and then remains basically unchanged. The presence of NP can reduce the frequency and PV. This reduction is strongly correlated with the values of WN and NP. At small WNs, the frequency and PV slightly increase in the presence of NP. Conversely, the frequency and PV significantly decrease with the increase in NP. In other words, the softening effect of NP shows an increasing trend as the WN increases. At the same WN, with the increase in NP, the softening effect of NP tends to decrease. The above analysis suggests that it is essential to consider the effect of nonlocal scale-dependent parameters on the dispersion curves of nanoplates. Neglecting the role of NP can lead to too large dispersion values, and these results are also supported by the experimental and molecular dynamics simulation data.

**Fig. 4:**
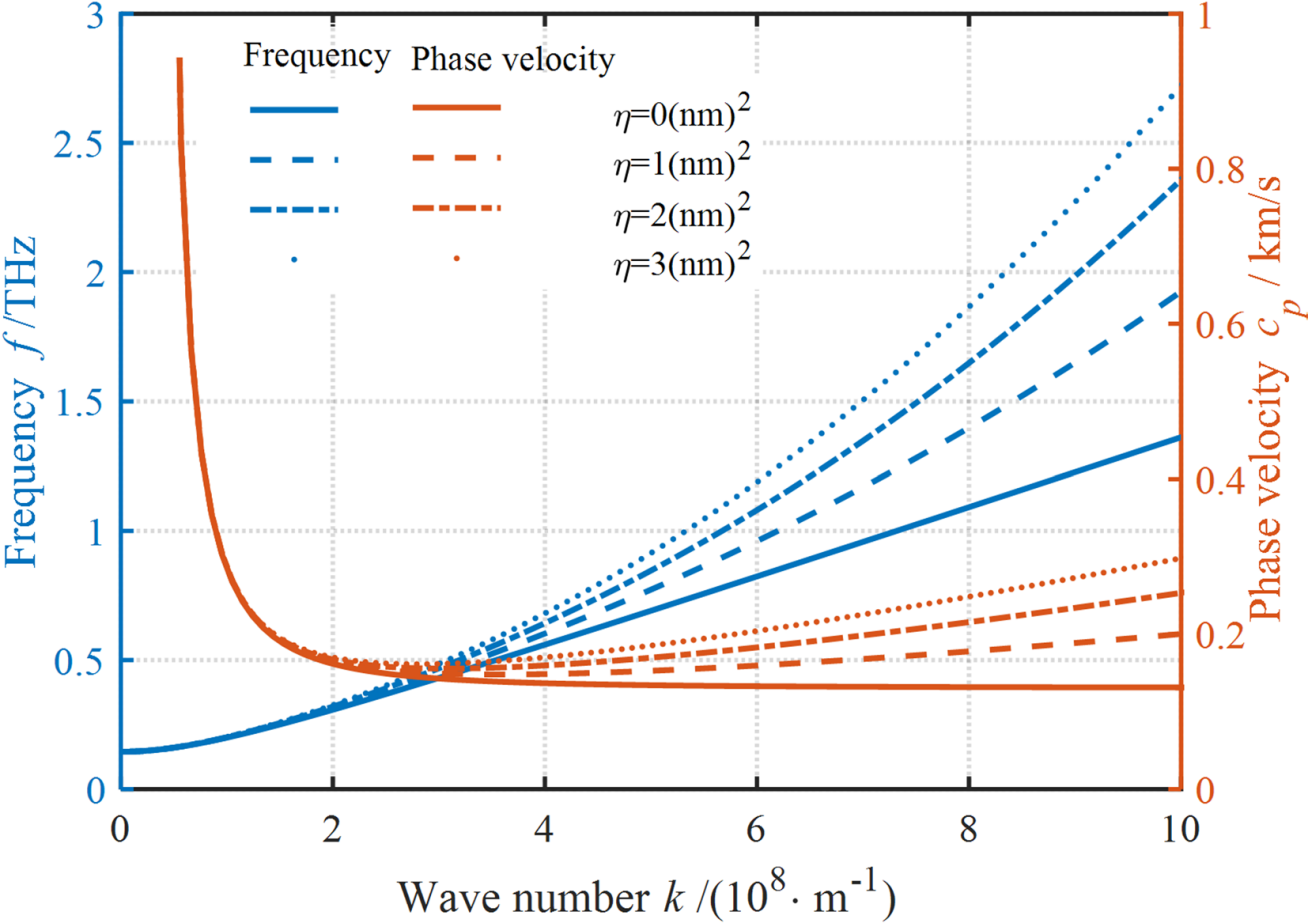
Effect of strain gradient fields on the dispersion curves.

Figure 4 shows that the strain gradient field has a significant effect on the dispersion curves. It’ s clear that neglecting the strain gradient field can also result in a near-linear increase in the frequency, while the PV rapidly decreases and then stabilizes. In contrast to the effect of nonlocal stress field on the frequency and PV, the presence of a strain gradient field causes an increase in frequency and PV. However, the effect of strain gradient field on the wave propagation characteristics is strongly correlated with the WN. The increase in LSP causes a negligible enhancement in the dispersion when the WN is small. Conversely, as the LSP increases, both the frequency and PV increase. Evidently, this hardening effect of the LSP progressively increases with the increase in WN. In addition, the hardening effect also increases with increase in LSP. Unlike the effect of nonlocal stress field on the dispersion curve, the increase in the LSP continues to have an enhancement effect on the dispersion curve, while the decrease in the NP has a gradual weakening effect on the dispersion curve. These results suggest that for examining the wave frequency dispersion properties of nanostructures, it is necessary to focus not only on the inner characteristic dimensions of the nanostructures but also on strain gradient fields acting on the structures.

**Fig. 5:**
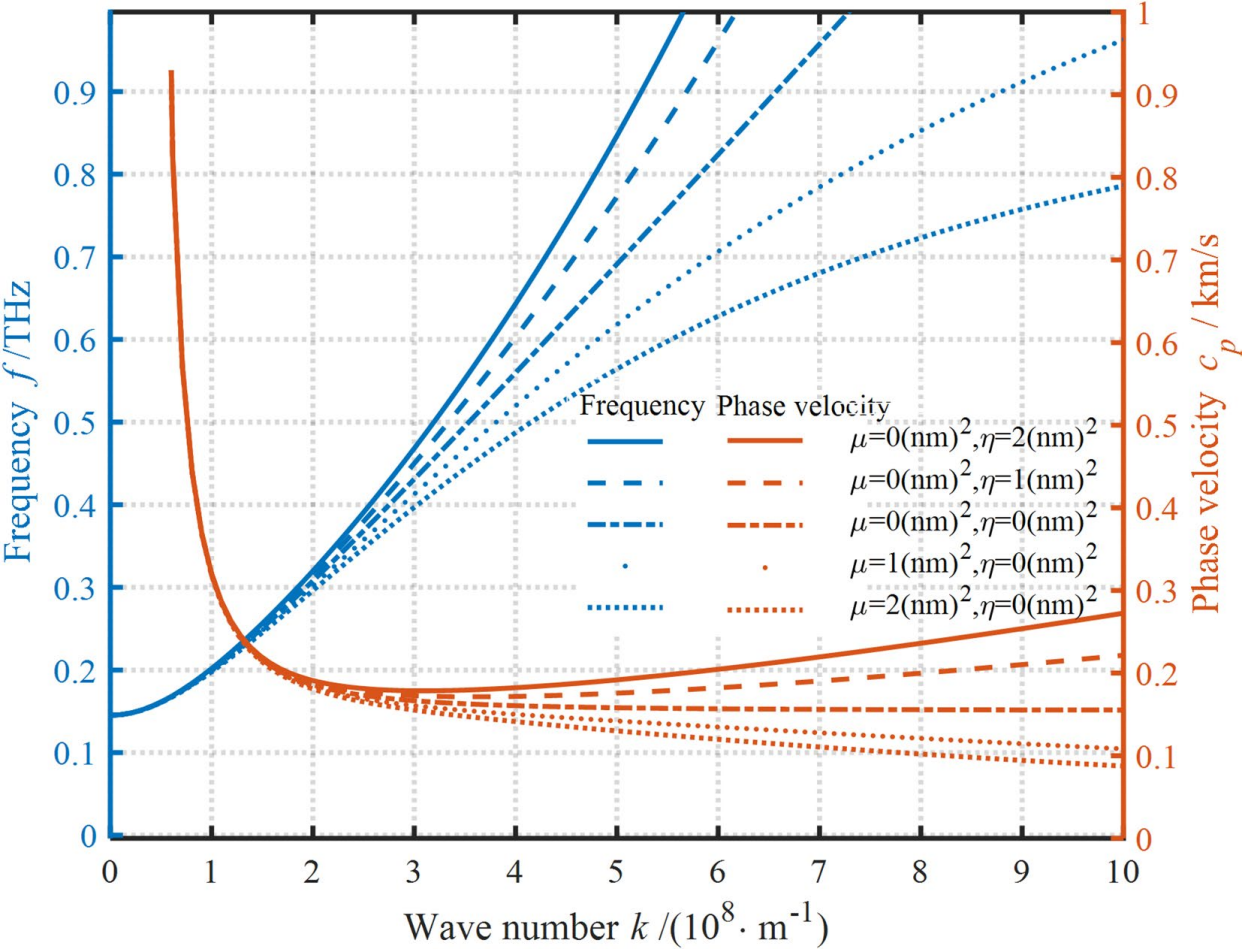
Effect of NSGT fields on the dispersion curves.

The effects of NP and LSP on the dispersion curves are presented in Fig. 5. It’s clear that if the effect of scale parameter is neglected, the frequency exhibits an approximately linear increase at large WNs, while the PV tends to approach a certain critical value. As established previously, both the WN and scale parameters exhibit a governing influence on dispersion curves. However, the scale parameter exerts negligible influence on dispersion behavior when the WN falls below a critical threshold, highlighting a regime-dependent interplay between these variables. As mentioned before, the WN and scale parameters have a crucial effect on the dispersion curves. The scale parameter has no impact on the dispersion curves when the WN is small. The reverse situation is dramatically different. It’s clear that when NP is larger than LSP, the softening effect is enhanced as the NP increases. However, when LSP is greater than NP, the hardening effect is promoted as the LSP increases.

Experimental studies have shown that the dispersive properties of nanostructures often exhibit both hardening and softening effects, and the inability of nonlocal stress and strain gradient theory to predict both hardening and softening effects has limited their application to only some specific nanomaterials. Therefore, the NSGT is suitable for capturing the scale-dependent properties of nanomaterials because it can simultaneously reflect both softening and hardening properties. Its greatest advantage lies in the fact that it can be fitted to obtain appropriate internal size parameters based on experimental and molecular dynamics simulations.

**Fig. 6:**
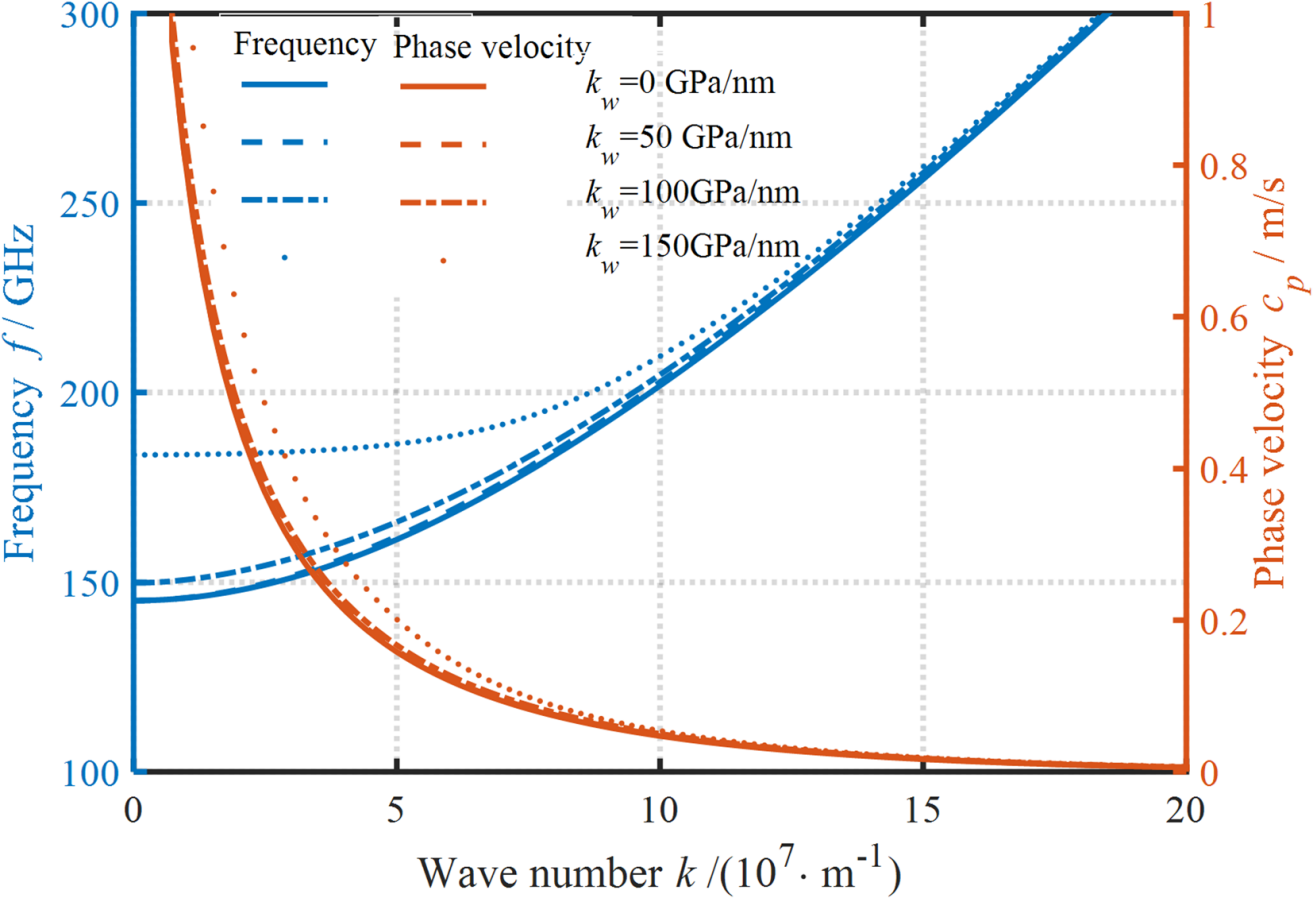
Effect of elasticity coefficients on the dispersion curves.

The influence of elasticity coefficient on the frequency and PV curves is presented in Fig. 6. Apparently, the effect of elasticity coefficient on the dispersion curve remains intricately connected to the WN. As the WN increases, the frequency and PV demonstrate substantially different variation trends. The effect of elasticity coefficient on the frequency is more pronounced at small WNs. With the increase in the elasticity coefficient, the frequency increases. It is important to mention that a uniform increase in the elasticity coefficient causes a non-uniform increase in the frequency. This implies that for substrate materials with large elasticity coefficients, the normal load needs to be carefully increased. Conversely, the increase in the elasticity coefficient at large WNs has a negligible effect on the frequency. This suggests that adjusting the elasticity coefficient to control the frequency change is ineffective. As with the frequency profile, the impact of elasticity coefficient on the PV is more pronounced at small WNs. Obviously, increasing the elasticity coefficient can induce an increase in the PV. This behavior arises from the enhanced normal stiffness of the nanoplate induced by a higher elasticity coefficient, which concurrently reveals that the substrate’s normal stiffness parameter exerts minimal influence on dispersion curve formation.

**Fig. 7:**
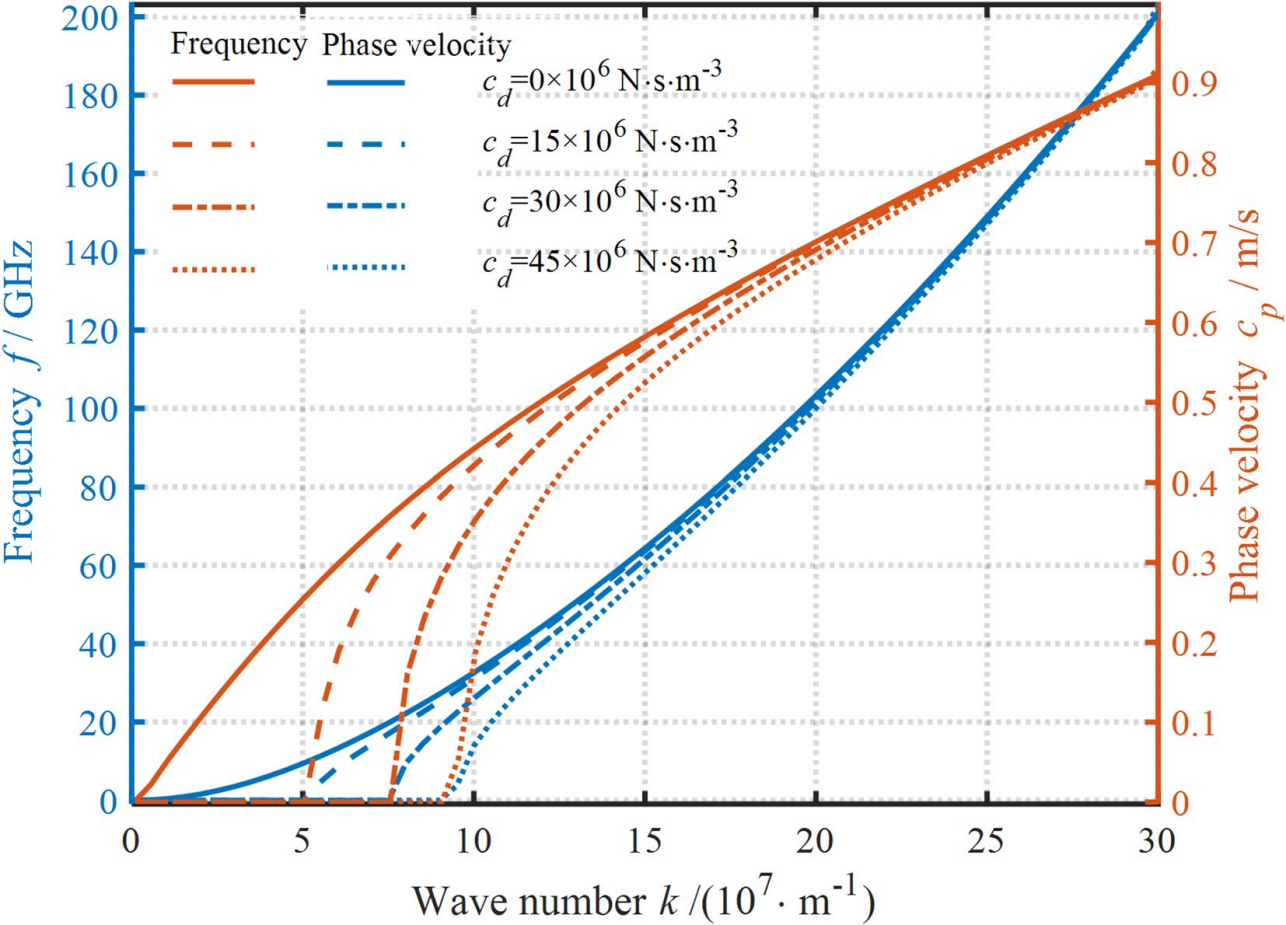
Impact of damping coefficients on the dispersion curves.

The impact of damping coefficients on the wave propagation behavior is displayed in Fig. 7. It is observed that the effect of damping coefficients on the dispersion curves varies considerably at different WNs. Specifically, both the frequency and the PV decrease as the damping coefficient increases at small WNs. This is mainly because the increase in the damping coefficient enhances the dynamic viscous properties of the nanoplates. By contrast, the damping coefficient has a relatively weaker effect on the dispersion curves at large WNs. This indicates that the obstruction of the wave transmission by the damping coefficient is reduced in this case. Notably, the presence of damping coefficients can potentially stop the wave propagation process. Further, an increase in the damping coefficient can delay this termination to larger WNs. In addition, this termination in the frequency and PV curves always occurs at the same WN. These phenomena indicate that the selection of a suitable viscous substrate material can stop the wave propagation at a specific frequency.

**Fig. 8:**
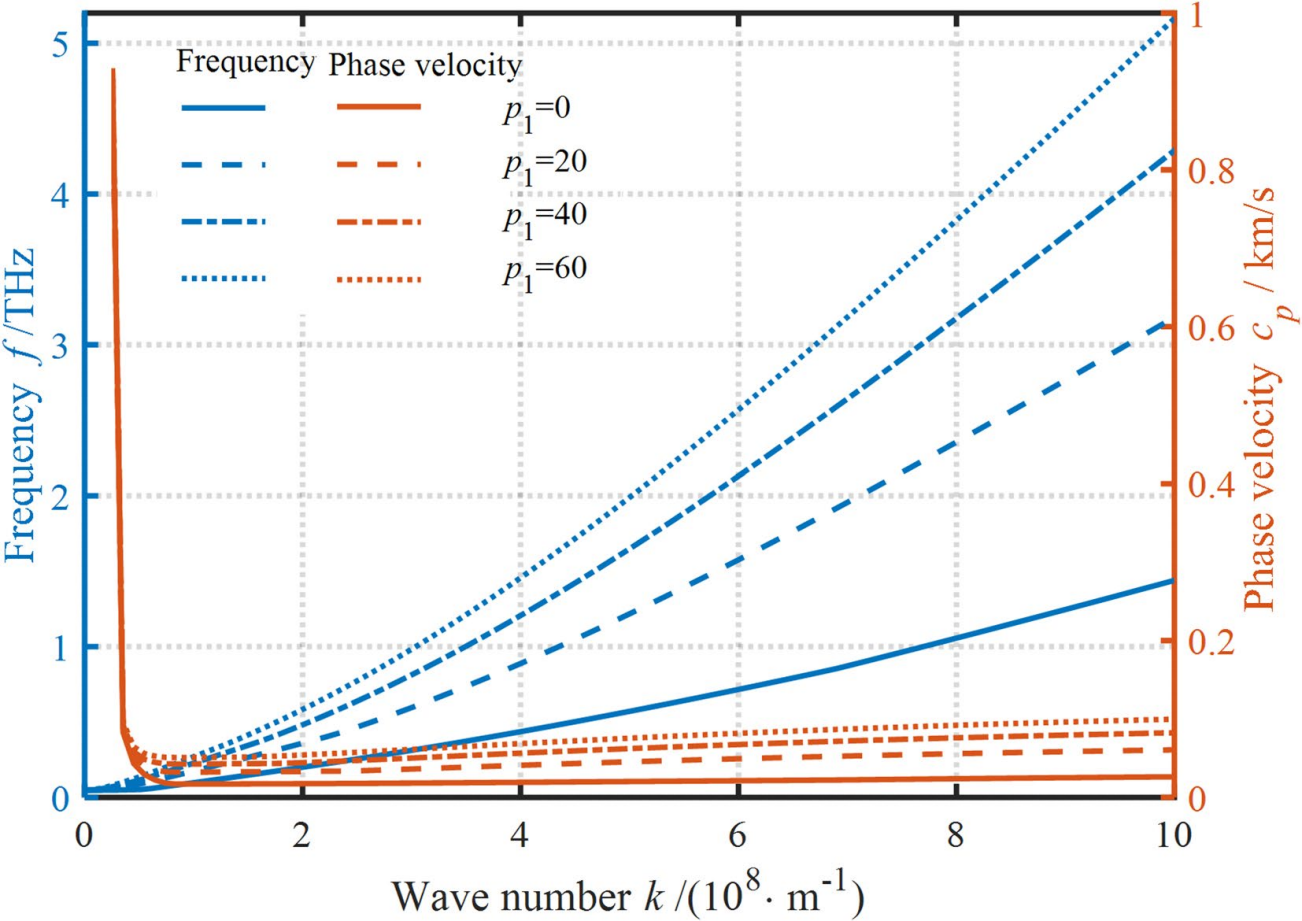
Effect of surface elasticity coefficient on the dispersion curve.

The effect of surface elasticity coefficient on the dispersion curve is shown in Fig. 8. It is evident that the impact of surface elasticity coefficient on the frequency and PV is governed by the WN. At small WNs, the dispersion curve is essentially independent of the surface elasticity coefficient. In other words, increasing the surface elasticity coefficient does not cause an increase in the frequency and PV. However, the impact of surface elasticity coefficient on the dispersion curves gradually strengthens as the WN increases. Further, at larger WN, an increase in the surface elasticity coefficient causes an increase in the frequency and PV, which is attributed to the improvement in the hardness of the nanoplate with the increase in the surface elasticity coefficient. The difference is that as the surface elasticity coefficient increases, the frequency grows rapidly, while the PV rises relatively slowly. It is evident that the identification of the surface elasticity parameters is crucial for exploring the dispersion characteristics of the nanoplates.

**Fig. 9:**
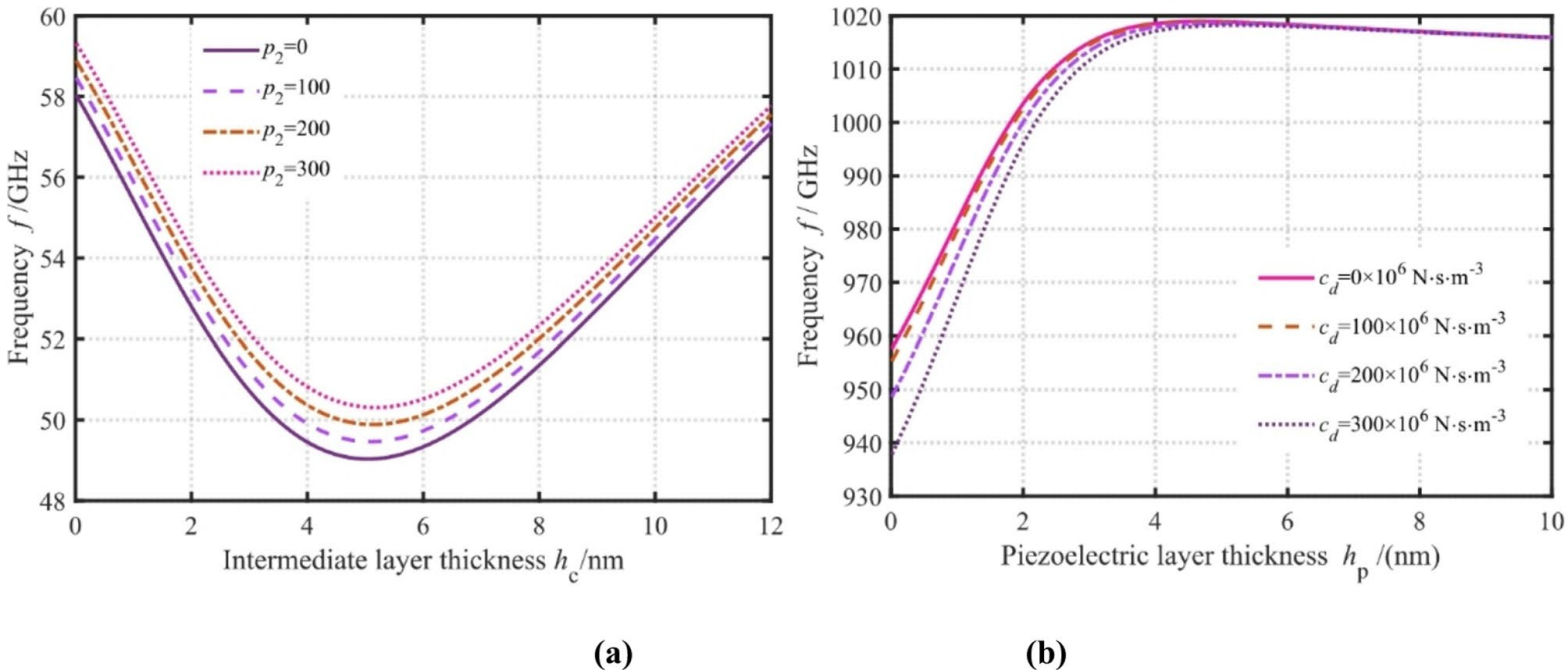
Effect of nanoplate thickness, surface piezoelectric coefficient, and damping coefficient on the frequency (**a** Intermediate layer thickness and surface piezoelectric coefficient, **b** Piezoelectric layer thickness and damping coefficient).

The influence of nanoplate thickness, surface piezoelectric coefficient, and damping coefficient on frequency is shown in Fig. 9. One can see that the impact of surface piezoelectric and damping coefficients on wave propagation is highly dependent on the thickness of the nanoplates. It is apparent that as the thickness of the intermediate metal layer increases, the nanoplate frequency first decreases and then increases. The decrease in the frequency is attributed to the predominance of scale effects, while the increase in frequency is attributed to the predominance of the higher elastic modulus of the metal layer. The frequency exhibits a proportional rise with increasing surface piezoelectric coefficient, attributed to the enhanced nanoplate stiffness induced by heightened electromechanical coupling. Notably, this influence becomes markedly pronounced as the metallic interlayer thickness approaches 5 nm, underscoring the critical interplay between surface piezoelectricity and geometric constraints in modulating dispersion behavior. These results indicate that frequency modulation can be achieved by adjusting the thickness of the metal layer. Further, the surface layer thickness and damping coefficient affect the wave propagation behavior, as shown in Fig. 9. With the increase in the piezoelectric layer thickness, the frequency rapidly increases, reaches the maximum value, and then decreases slowly. This is ascribed to the competition between the enhancement effect of piezoelectric elastic modulus on the frequency and the weakening effect of its thickness on the frequency. It is also observed that the frequency decreases due to the increase in the damping coefficient, and this effect is most prominent when the piezoelectric layer thickness is small. This effect weakens when the thickness increases, and eventually the damping coefficient has no effect on the frequency. This implies that the effect of piezoelectric layer thickness need not be considered if the frequency is appropriately adjusted by the damping factor.

**Fig. 10:**
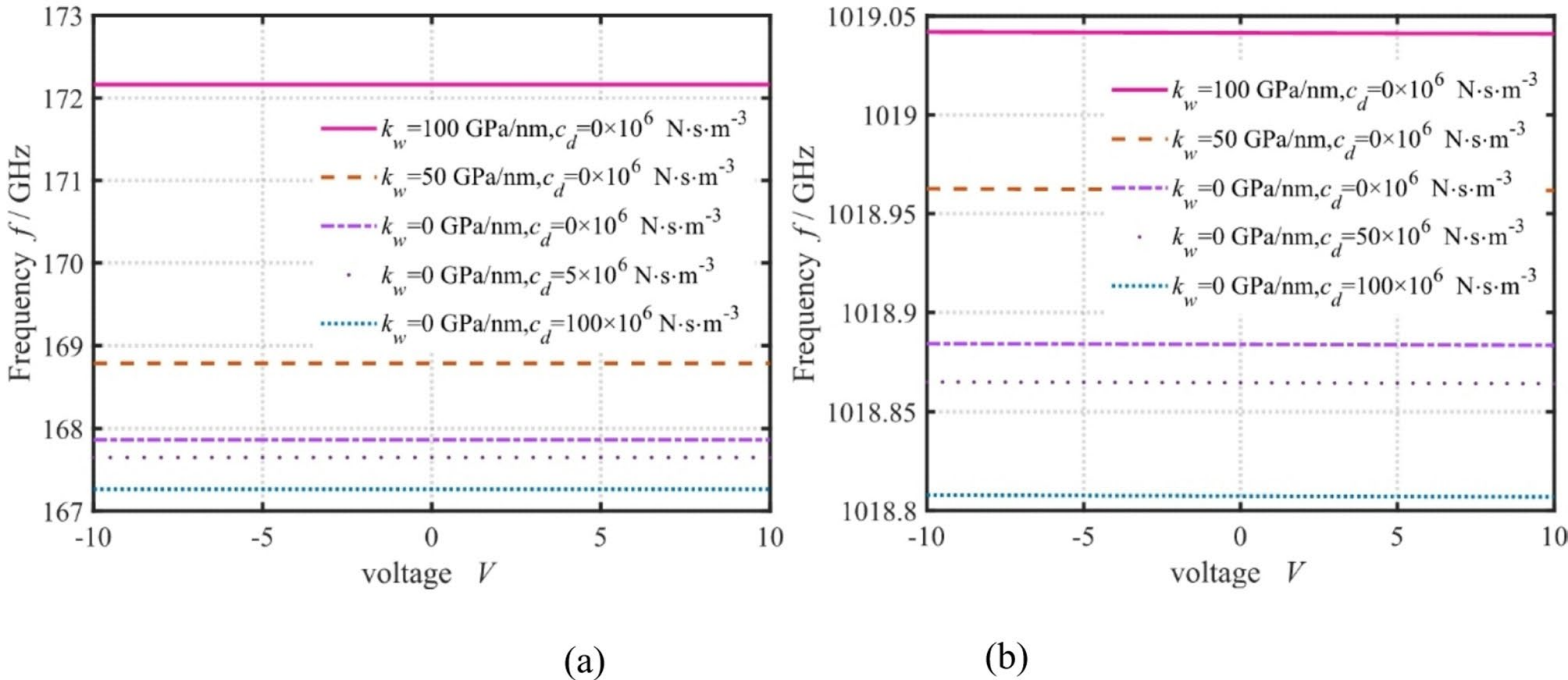
Effect of voltage, elasticity, and damping coefficients on the frequency for different WNs (**a** *k* = 10^7^, **b** *k* = 10^8^).

The effects of voltage, elasticity, and damping coefficients on the frequency are shown in Fig. 10. It’s clear that increasing the voltage slightly affects the frequency. This implies that the piezoelectric sandwich nanoplates have good voltage stability, which means that the structure is suitable for use in devices with high voltage drift requirements. Under different WNs, the frequency increases with the increase in the elasticity coefficient, while it decreases with the increase in the damping coefficient. This is because the increase in the elasticity coefficient enhances the normal stiffness of the nanoplates, while the increase in the damping coefficient enhances the viscous properties of the nanoplates. However, the effects of elasticity and damping coefficients on the frequency are inconsistent for different WNs. The elasticity and damping coefficients have a more prominent effect on the frequency at smaller WNs. They have a much weaker effect on the frequency at larger WNs. These phenomena suggest that to regulate the frequency of nanoplates through elasticity and damping coefficients, the role of WN must be considered.

**Fig. 11:**
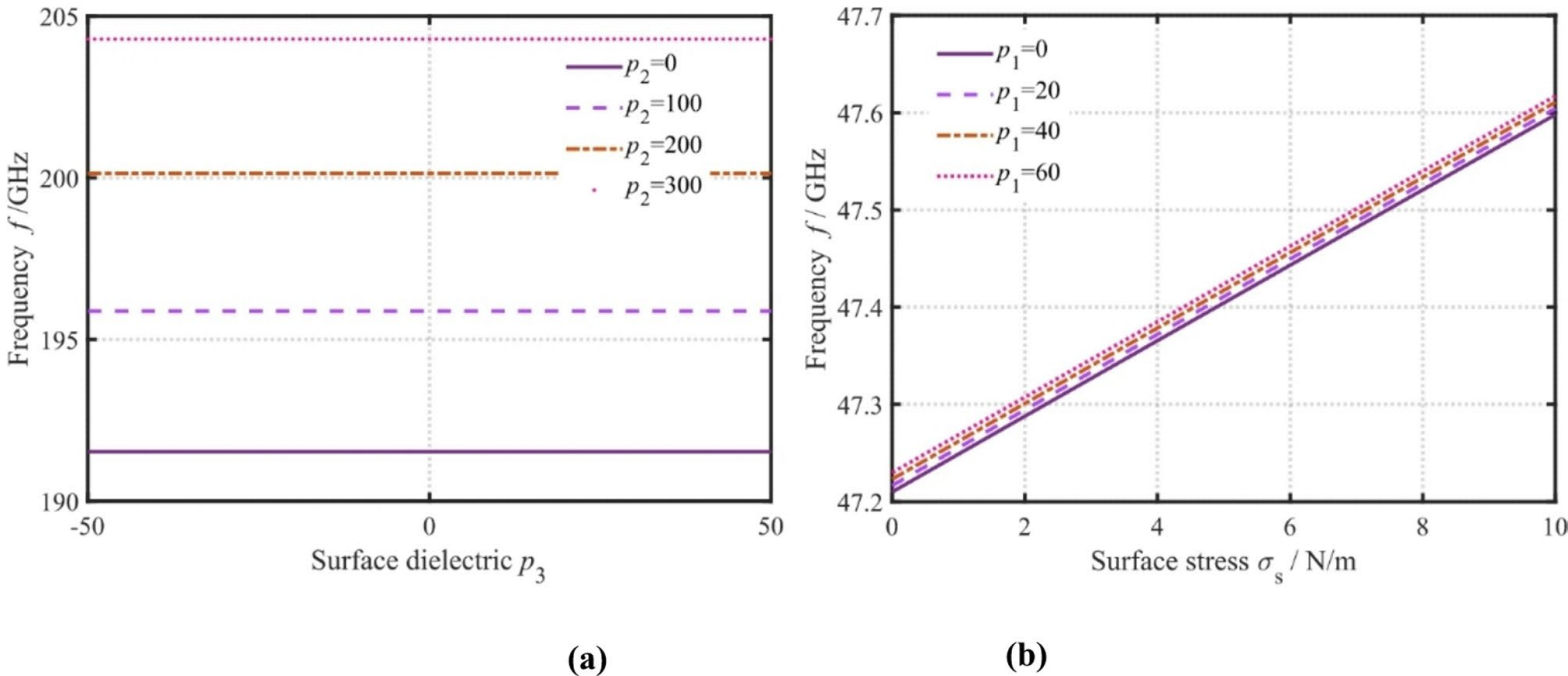
Effect of surface stress, surface elasticity, surface piezoelectricity, and surface dielectric coefficient on the frequency(a: surface piezoelectricity, and surface dielectric coefficient, b:surface stress, surface elasticity).

The effects of surface parameters, including surface stress, surface elasticity, surface piezoelectricity, and surface dielectric coefficient, on the frequency are demonstrated in Fig. 11. It is evident that as the surface piezoelectric coefficient increases, the frequency increases. However, with the increase in the surface dielectric coefficient, the frequency remains almost constant. This is because the surface piezoelectricity affects the stiffness of the nanoplates, while the surface dielectric coefficient has a minor effect on the stiffness. Furthermore, the effects of surface residual stress and surface elasticity coefficient on the wave propagation characteristics are displayed in Fig. 11. The frequency exhibits a pronounced elevation with increasing surface stress and surface elasticity coefficients. This trend is governed by two synergistic mechanisms: (1) the surface elasticity coefficient directly enhances nanoplate stiffness, and (2) surface stress amplifies tensile forces, further stiffening the structure. These findings underscore the necessity to holistically account for surface stress, surface elasticity coefficients, and surface piezoelectric coefficients when analyzing dispersion characteristics in nanostructured systems.

## Conclusions

The effects of NP and LSP on the dispersion curves and the impact of surface elasticity, surface piezoelectric coefficient, and surface dielectric parameter on the frequency of piezoelectric sandwich nanoplates were explored. Further, the coupled effect of viscoelastic parameters and WN on the dispersion curves was examined. The main results of the study are summarized as follows:

(1) The NP and LSP had softening and hardening effects on the dispersion curves, respectively, and these effects became more pronounced at larger WNs. Further, the effects were canceled out when the NP and LSP were equal. The dispersion curves are governed by synergistic scale dependencies and wavenumber coupling, reflecting the interplay of geometric and wave-propagation dynamics in nanostructured systems.
(2) The elasticity coefficient had a local and nonlinear effect on the dispersion curves, which was closely related to the WN. The dispersion enhancement was more pronounced at smaller WNs. An increase in the damping factor could cause the wave propagation to stop. Further, the frequency curve and the PV curve terminated at the same time. This property of damping coefficient suggested that the viscous properties of the material should be considered in the selection of foundation. The frequency evolution is governed by a synergistic interplay of viscoelasticity, geometric thickness, applied voltage, and wavenumber, underscoring a multiparametric dependence in dynamic response.
(3) Both surface elasticity and surface piezoelectricity contributed to the enhancement of the dispersion curves, and the surface piezoelectricity, damping, and nanoplate thickness had a coupled effect on the frequency. The impact of elasticity and damping coefficients on the frequency was also governed by the WN. The surface dielectric parameter had a relatively minor effect on the frequency, and the increase in surface stress enhanced the frequency. The dispersion characteristics of nanoplates necessitate the incorporation of surface effects.

## Data Availability

The datasets used and analysed during the current study can be obtained from the corresponding author. The email address of the corresponding author is 55192473@qq.com.

## References

[1] Rai, S. & Gupta, A. Dynamic response of sandwich functionally graded nanoplate under thermal environments and elastic foundations using dynamic stiffness method. *Sci. Rep.* **14**(1), 21689. 10.1038/s41598-024-70210-2 (2024). 39289400 10.1038/s41598-024-70210-2 PMC11408604

[2] Li, Y., Li, Y. Q., Guo, Z., Wang, H. & Wang, C. D. Band gaps of elastic waves in 1-D dielectric phononic crystal with the flexoelectric and strain gradient effects consideration. *Sci. Rep.* **14**(1), 24035. 10.1038/s41598-024-75049-1 (2024). 39402096 10.1038/s41598-024-75049-1 PMC11479263

[3] Aljadani, M. H. The porosity effect on the buckling analysis of functionally graded plates under thermal environment using a Quasi-3D theory. *Sci. Rep.* **14**(1), 30216. 10.1038/s41598-024-79894-y (2024). 39632959 10.1038/s41598-024-79894-y PMC11618368

[4] Youssef, A. A., Amein, N. K., Salama, F. A., Ghaleb, A. F. & Ahmed, E. A. A. Nonlinear Rayleigh wave propagation in a three-layer sandwich structure in dual-phase-lag. *Sci. Rep.* **14**(1), 27134. 10.1038/s41598-024-73912-9 (2024). 39511216 10.1038/s41598-024-73912-9 PMC11543657

[5] Chu, L. L., Dui, G. S. & Ju, C. J. Flexoelectric effect on the bending and vibration responses of functionally graded piezoelectric nanobeams based on general modified strain gradient theory. *Compos. Struct.* **186**, 39–49. 10.1016/j.compstruct.2017.10.083 (2018).

[6] Tao, H. M. et al. Size-dependent free vibration analysis of honeycomb sandwich microplates integrated with piezoelectric actuators based on the modified strain gradient theory. *Compos. Struct.* **305**, 116555. 10.1016/J.COMPSTRUCT.2022.116555 (2023).

[7] Vahid, M., Mohammad, M. K., Younes, M. & Ebrahimi, F. Modified strain gradient theory for nonlinear vibration analysis of functionally graded piezoelectric doubly curved microshells. *P I Mech. Eng. C-***236**(8), 4219–4231. 10.1177/09544062211045886 (2022).

[8] Alfurjan, M. S. H. et al. Dynamic instability of nanocomposite piezoelectri-leptadenia pyrotechnica rheological elastomer-porous functionally graded materials micro viscoelastic beams at various strain gradient higher-order theories. *Polym. Compos.* **43**(1), 282–298. 10.1002/PC.26373 (2021).

[9] Lim, C. W., Zhang, G. & Reddy, J. N. A higher-order nonlocal elasticity and strain gradient theory and its applications in wave propagation. *J. Mech. Phys. Solids*. **78**, 298–313. 10.1016/j.jmps.2015.02.001 (2015).

[10] Zeng, S., Wang, K. F., Wang, B. L. & Wu, J. W. Vibration analysis of piezoelectric sandwich nanobeam with flexoelectricity based on nonlocal strain gradient theory. *Appl. Math. Mech.* **41**, 859–880. 10.1007/s10483-020-2620-8 (2020).

[11] Fateme, S. & Majid, G. Investigation of flexoelectric effect on nonlinear vibration and dynamic instability of piezoelectric sandwich micro/nanobeam using the nonlocal strain gradient theory. *Int. J. Struct. Stab. Dy*. **23**(04), 2350045. 10.1142/S0219455423500451 (2023).

[12] Zhou, S. S. et al. Electro-mechanical responses of transversely isotropic piezoelectric nano-plate based on the nonlocal strain gradient theory with flexoelectric effect. *Acta Mech.* **234**(11), 5647–5672. 10.1007/S00707-023-03690-4 (2023).

[13] Sharifi, Z., Khordad, R., Gharaati, A. & Forozani, G. An analytical study of vibration in functionally graded piezoelectric nanoplates: nonlocal strain gradient theory. *Appl. Math. Mech.* **40**(12), 1723–1740. 10.1007/s10483-019-2545-8 (2019).

[14] Zhang, L., Guo, J. H. & Xing, Y. M. Bending analysis of functionally graded one-dimensional hexagonal piezoelectric quasicrystal multilayered simply supported nanoplates based on nonlocal strain gradient theory. *Acta Mech. Solida Sin*. **34**, 237–251. 10.1007/s10338-020-00204-w (2021).

[15] Amiri, A., Talebitooti, R. & Li, L. Wave propagation in viscous-fluid-conveying piezoelectric nanotubes considering surface stress effects and Knudsen number based on nonlocal strain gradient theory. *Eur. Phys. J. Plus*. **133**(7), 1–17. 10.1140/epjp/i2018-12077-y (2018).

[16] Al-Furjan, M. S. H., Dehini, R., Khorami, M., Habibi, M. & Jung, D. W. On the dynamics of the ultra-fast rotating cantilever orthotropic piezoelectric nanodisk based on nonlocal strain gradient theory. *Compos. Struct.* **255**, 112990. 10.1016/j.compstruct.2020.112990 (2021).

[17] Arefi, M., Pourjamshidian, M. & Arani, G. A. Dynamic instability region analysis of sandwich piezoelectric nano-beam with FG-CNTRCs face-sheets based on various high-order shear deformation and nonlocal strain gradient theory. *Steel Compos. Struct.* **32**(2), 151–171. 10.12989/scs.2019.32.2.151 (2019).

[18] Ebrahimi, F., Karimiasl, M. & Mahesh, V. Nonlocal and surface effects on the bending analysis of flexoelectrically actuated piezoelectric microbeams in hygrothermal environment. *Sadhana-Acad P Eng. S*. **46**(2), 107. 10.1007/S12046-021-01625-0 (2021).

[19] Ebrahimi, F. & Barati, M. R. Magnetic field effects on buckling characteristics of smart flexoelectrically actuated piezoelectric nanobeams based on nonlocal and surface elasticity theories. *Microsyst. Technol.* **24**(5), 2147–2157. 10.1007/s00542-017-3652-x (2018).

[20] Fathi, M. & Ghassemi, A. The effects of surface stress and nonlocal small scale on the uniaxial and biaxial buckling of the rectangular piezoelectric nanoplate based on the two variable-refined plate theory. *J. Braz Soc. Mech. Sci.* **39**(8), 3203–3216. 10.1007/s40430-017-0817-6 (2017).

[21] Sun, J., Wang, Z. Y., Zhou, Z. H., Xu, X. S. & Lim, C. W. Surface effects on the buckling behaviors of piezoelectric cylindrical nanoshells using nonlocal continuum model. *Appl. Math. Model.* **59**, 341–356. 10.1016/j.apm.2018.01.032 (2018).

[22] Kachapi, S. H. H., Daniali, H. M., Dardel, M. & Fathi, A. The effects of nonlocal and surface/interface parameters on nonlinear vibrations of piezoelectric nanoresonator. *J. Intell. Mater. Syst. Struct.* **31**(6), 818–842. 10.1177/1045389X19898756 (2020).

[23] Wang, W. J., Li, P., Jin, F. & Ji, W. Vibration analysis of piezoelectric ceramic circular nanoplates considering surface and nonlocal effects. *Compos. Struct.* **140**, 758–775. 10.1016/j.compstruct.2016.01.035 (2016).

[24] Ebrahimi, F. & Barati, R. M. Thermo-mechanical vibration analysis of nonlocal flexoelectric/piezoelectric beams incorporating surface effects. *Struct. Eng. Mech.* **65**(4), 435–445. 10.12989/sem.2018.65.4.435 (2018).

[25] Hosseini-Hashemi, S., Nahas, I., Fakher, M. & Nazemnezhad, R. Surface effects on free vibration of piezoelectric functionally graded nanobeams using nonlocal elasticity. *Acta Mech.* **225**(6), 1555–1564. 10.1007/s00707-013-1014-z (2014).

[26] Salman, E. N. & Mahya, B. Comprehensive nonlocal analysis of piezoelectric nanobeams with surface effects in bending, buckling and vibrations under magneto-electro-thermo-mechanical loading. *Mater. Res. Express*. **5**(3), 035028. 10.1088/2053-1591/aab46d (2018).

[27] Dhua, S., Mondal, S. & Maji, A. Surface effects on wave propagation in piezoelectric–piezomagnetic loosely bonded bilayer system using nonlocal theory of elasticity. *Thin Wall Struct.* **197**, 111612. 10.1016/J.TWS.2024.111612 (2024).

[28] Zhang, Y. W. et al. Surface and thermal effects of the flexural wave propagation of piezoelectric functionally graded nanobeam using nonlocal elasticity. *Comp. Mater. Sci.* **97**, 222–226. 10.1016/j.commatsci.2014.10.046 (2015).

[29] Zhang, L. L., Liu, J. X., Fang, X. Q. & Nie, G. Q. Effects of surface piezoelectricity and nonlocal scale on wave propagation in piezoelectric nanoplates. *Eur. J. Mech. A-Solid*. **46**, 22–29. 10.1016/j.euromechsol.2014.01.005 (2014).

[30] Ebrahimi, F. & Dabbagh, A. Wave propagation analysis of embedded nanoplates based on a nonlocal strain gradient-based surface piezoelectricity theory. *Eur. Phys. J. Plus*. **132**(11), 449. 10.1140/epjp/i2017-11694-2 (2017).

[31] Khabaz, M. K., Eftekhari, S. A. & Toghraie, D. Vibration and dynamic analysis of a cantilever sandwich microbeam integrated with piezoelectric layers based on strain gradient theory and surface effects. *Appl. Math. Comput.* **419**, 126867. 10.1016/J.AMC.2021.126867 (2022).

[32] Mohammadimehr, M., Navi, R. B. & Arani, G. A. Dynamic stability of modified strain gradient theory sinusoidal viscoelastic piezoelectric polymeric functionally graded single-walled carbon nanotubes reinforced nanocomposite plate considering surface stress and agglomeration effects under hydro-thermo-electro-magneto-mechanical loadings. *Mech. Adv. Mater. Struct* **24**(16), 1325–1342. 10.1080/15376494.2016.1227507 (2017).

[33] Ansari, R., Nesarhosseini, S., Faraji, O. M. & Rouhi, H. Size-dependent buckling analysis of piezoelectric nanobeams resting on elastic foundation considering flexoelectricity effect using the stress-driven nonlocal model. *Eur. Phys. J. Plus*. **136**(8), 876. 10.1140/EPJP/S13360-021-01837-7 (2021).

[34] Fatemeh, A. & Hadi, A. Thermo-electro-mechanical buckling analysis of sandwich nanocomposite microplates reinforced with graphene platelets integrated with piezoelectric face sheets resting on elastic foundation. *Comput. Math. Appl.* **101**, 38–50. 10.1016/J.CAMWA.2021.09.009 (2021).

[35] Yang, Y. K., Dong, Y. H. & Li, Y. H. Buckling of piezoelectric sandwich microplates with arbitrary in-plane BCs rested on foundation: effect of hygro-thermo-electro-elastic field. *Eur. Phys. J. Plus*. **135**(7), 1–20. 10.1140/epjp/s13360-020-00098-0 (2020).

[36] Zenkour, A. M. & Alghanmi, R. A. Static response of sandwich plates with FG core and piezoelectric faces under thermo-electro-mechanical loads and resting on elastic foundations. *Thin Wall Struct.* **157**, 107025. 10.1016/j.tws.2020.107025 (2020).

[37] Wang, W., Li, H. N. & Yao, L. Q. Static bending and vibration analysis of a rectangular functionally gradient piezoelectric plate on an elastic foundation. *Appl. Sci.* **12**(3), 1517. 10.3390/APP12031517 (2022).

[38] Hong, T. N. Free vibration and static bending analysis of piezoelectric functionally graded material plates resting on one area of two-parameter elastic foundation. *Math. Probl. Eng.* **2020**, 9236538. 10.1155/2020/9236538 (2020).

[39] Morteza, B., Saeed, J. M., Hamid, M. M. & Hassan, F. Free vibration behavior of an elliptical sandwich microplate, consisting of a saturated porous core and two piezoelectric face layers, standing on an elastic foundation. *Acta Mech.* **233**(8), 3253–3290. 10.1007/S00707-022-03227-1 (2022).

[40] Minh, P. P. & Nguyen, T. V. On the vibration analysis of rotating piezoelectric functionally graded beams resting on elastic foundation with a higher-order theory. *Int. J. Aerosp. Eng.* **2022**, 9998691. 10.1155/2022/9998691 (2022).

[41] Al-Furjan, M. S. H., Farrokhian, A., Keshtegar, B., Kolahchi, R. & Trung, N. T. Dynamic stability control of viscoelastic nanocomposite piezoelectric sandwich beams resting on Kerr foundation based on exponential piezoelasticity theory. *Eur. J. Mech. A-Solid*. **86**, 104169. 10.1016/j.euromechsol.2020.104169 (2021).

[42] Yassine, K. E., Ahmed, A., Omar, O., Said, R. & Rhali, B. An analytical approach to geometrically nonlinear free and forced vibration of piezoelectric functional gradient beams resting on elastic foundations in thermal environments. *Mech. Adv. Mater. Struc*. **30**(1), 131–143. 10.1080/15376494.2021.2009601 (2021).

[43] Luo, T. X. et al. Scale effect on the nonlinear vibration of piezoelectric sandwich nanobeams on Winkler foundation. *J. Vib. Eng. Technol.* **9**(6), 1289–1303. 10.1007/S42417-021-00297-8 (2021).

[44] Wang, Y. Q., Liu, Y. F. & Yang, T. H. Nonlinear thermo-electro-mechanical vibration of functionally graded piezoelectric nanoshells on winkler–pasternak foundations via nonlocal Donnell’s nonlinear shell theory. *Int. J. Struct. Stab. Dy*. **19**(9), 1950100. 10.1142/S0219455419501001 (2019).

[45] Reda, A. et al. Surface stress effect on nonlinear dynamical performance of nanobeam-type piezoelectric energy harvesters via meshless collocation technique. *Eng. Anal. Bound. Elem.* **152**, 104–119. 10.1016/j.enganabound.2023.04.003 (2023).

[46] Shahzad, A. M. et al. On the role of surface stress tensor in the nonlinear response of time-dependent mechanical actuated nanoplate-type energy piezo-harvesters. *Mech. Adv. Mater. Struct.* **31**(26), 8113–8135. 10.1080/15376494.2023.2254757 (2024).

[47] Sahmani, S., Safaei, B. & Fan, F. Nonlinear dynamical response of sinusoidal impulsive actuated piezoelectric/porous sandwich nanoharvesters via GM-based Meshfree collocation formulations. *Comput. Struct.* 299107389. 10.1016/j.compstruc.2024.107389 (2024).

[48] Saeed, S., Alireza, A., Paria, T. & Rajabi, M. Z. Vibration analysis of piezoelectric carbon nanotube considering surface effects, located in the magnetic field and resting on nonlinear viscoelastic foundation. *Nanobiotechnol. Rep.* **17**(1), 64–73. 10.1134/S2635167622010141 (2022).

[49] Azhdarzadeh, M., Jahangiri, R., Allahverdizadeh, A., Dadashzadeh, B. & Nabatiet, R. Investigation of nonlinear thermo-elastic behavior of fluid conveying piezoelectric microtube reinforced by functionally distributed carbon nanotubes on viscoelastic-hetenyi foundation. *Eur. J. Comput. Mech.* **31**(1), 65–100. 10.13052/EJCM2642-2085.3113 (2022).

[50] Zhang, D. P., Liu, M. W., Wang, Z. X. & Lei, Y. J. Thermo-electro-mechanical vibration of piezoelectric nanobeams resting on a viscoelastic foundation. *J. Phys: Conf. Ser.* **1759**(1), 012029. 10.1088/1742-6596/1759/1/012029 (2021).

[51] Zhang, D. P., Lei, Y. J. & Shen, Z. B. Thermo-electro-mechanical vibration analysis of piezoelectric nanoplates resting on viscoelastic foundation with various boundary conditions. *Int. J. Mech. Sci.* **131–132**, 1001–1015. 10.1016/j.ijmecsci.2017.08.031 (2017).

[52] Wang, X. T., Liu, J., Hu, B., Li, Z. N. & Zhang, B. Wave propagation in porous functionally graded piezoelectric nanoshells resting on a viscoelastic foundation. *Phys. E*. **151**, 115615. 10.1016/J.PHYSE.2022.115615 (2023).

[53] Li, Z. N., Liu, J., Hu, B., Wang, Y. X. & Shen, H. M. Wave propagation analysis of porous functionally graded piezoelectric nanoplates with a visco-Pasternak foundation. *Appl. Math. Mech.* **44**(1), 35–52. 10.1007/S10483-023-2953-7 (2022).

[54] Tong, H. L., Wen, B. Q., Xiang, Y., Lei, Z. X. & Lim, C. W. Elastic buckling of nanoplates based on general third-order shear deformable plate theory including both size effects and surface effects. *Int. J. Mech. Mater. Des.* **17**(3), 1–23. 10.1007/S10999-021-09545-X (2021).38624689

[55] Chen, W. Q., Ying, J., Cai, J. B. & Ye, G. R. Benchmark solution of imperfect angle-ply laminated rectangular plates in cylindrical bending with surface piezoelectric layers as actuator and sensor. *Comput. Struct.* **82**(22), 1773–1784. 10.1016/j.compstruc.2004.05.011 (2004).

[56] Gghorbanpour-Arani, A., Kolahdouzan, F. & Abdollahian, M. Nonlocal buckling of embedded magnetoelectroelastic sandwich nanoplate using refined zigzag theory. *Appl. Math. Mech.* **39**(04), 529–546. 10.1007/s10483-018-2319-8 (2018).

